# Simulating the Effects of Sea Level Rise on the Resilience and Migration of Tidal Wetlands along the Hudson River

**DOI:** 10.1371/journal.pone.0152437

**Published:** 2016-04-04

**Authors:** Nava M. Tabak, Magdeline Laba, Sacha Spector

**Affiliations:** 1 Conservation Science Department, Scenic Hudson, Inc., Poughkeepsie, New York, United States of America; 2 School of Integrative Plant Sciences, Section of Soil & Crop Sciences, Cornell University, Ithaca, New York, United States of America; University of Sydney, AUSTRALIA

## Abstract

Sea Level Rise (SLR) caused by climate change is impacting coastal wetlands around the globe. Due to their distinctive biophysical characteristics and unique plant communities, freshwater tidal wetlands are expected to exhibit a different response to SLR as compared with the better studied salt marshes. In this study we employed the Sea Level Affecting Marshes Model (SLAMM), which simulates regional- or local-scale changes in tidal wetland habitats in response to SLR, and adapted it for application in a freshwater-dominated tidal river system, the Hudson River Estuary. Using regionally-specific estimated ranges of SLR and accretion rates, we produced simulations for a spectrum of possible future wetland distributions and quantified the projected wetland resilience, migration or loss in the HRE through the end of the 21^st^ century. Projections of total wetland extent and migration were more strongly determined by the rate of SLR than the rate of accretion. Surprisingly, an increase in net tidal wetland area was projected under all scenarios, with newly-formed tidal wetlands expected to comprise at least 33% of the HRE’s wetland area by year 2100. Model simulations with high rates of SLR and/or low rates of accretion resulted in broad shifts in wetland composition with widespread conversion of high marsh habitat to low marsh, tidal flat or permanent inundation. Wetland expansion and resilience were not equally distributed through the estuary, with just three of 48 primary wetland areas encompassing >50% of projected new wetland by the year 2100. Our results open an avenue for improving predictive models of the response of freshwater tidal wetlands to sea level rise, and broadly inform the planning of conservation measures of this critical resource in the Hudson River Estuary.

## Introduction

Tidal wetlands are among the most productive yet highly vulnerable ecosystems in the world [1–4]. Among an array of ecological functions, these diverse ecosystems provide services such as nutrient cycle regulation, water filtration, protection from coastal storms, and fish and wildlife habitat [2,5–9]. Their location within or near the range of daily tides drives the notable productivity and unique ecosystem functions of tidal wetlands [2,10], but also exposes them to climate change impacts from accelerating rates of Sea Level Rise (SLR), as currently being observed and projected by models from various parts of the world [11–15]. To maintain the ecological function of tidal wetlands and their benefits to a society that is increasingly living along shores [16,17], it is essential to be able to forecast the magnitude and nature of tidal wetland ecosystem responses to SLR.

Coastal managers and researchers of estuarine ecosystems have a keen need to understand the adaptive capacity of tidal wetlands in response to SLR, and recent studies have documented a range of ongoing and potential impacts of SLR on coastal wetlands across the globe, including losses of wetland area, composition shifts, and/or changes in resiliency [12,18–25]. Most efforts to date have focused on systems dominated by brackish and saltwater wetlands, and a body of literature on simulating the impacts of SLR on these systems has been developed [18,20,22,25–27]. Similar efforts to simulate the impacts of SLR on freshwater tidal wetlands along tidal river systems have not been widely attempted or published.

The tidal system of the Hudson River Estuary (HRE) in New York State, USA, is uncommon in that approximately 80% of the wetland area experiences strong tidal influence with limited or no saltwater intrusion, and is classified as freshwater tidal wetland [28,29]. Freshwater tidal wetlands have been relatively understudied, but their distinctness and importance have led to greater interest and efforts in recent years [6,30]. The tidal wetlands of the HRE are recognized for their ecological, aesthetic and economic contributions to a region where high biodiversity co-occurs with relatively dense human habitation. The freshwater wetlands of the HRE represent one of the largest concentrations of these habitats along the northeastern United States Atlantic seaboard [9,31].

In this study we employed the Sea Level Affecting Marshes Model (SLAMM), which is designed to simulate regional- or local-scale changes in tidal wetland habitats in response to SLR [32,33], and adapted it for application in a freshwater-dominated tidal river system. Using regionally-specific wetland maps, SLR projections, and accretion rates, we produced simulations of future tidal wetland distributions and quantified the likely resilience, migration, or loss of tidal wetland areas in the HRE under a range of future scenarios. This information can be used for estuary-wide resilience planning and to prioritize conservation, mitigation, restoration, and further study efforts among the many wetland sites in the estuary.

## Materials and Methods

### Study Site

**Fig 1:**
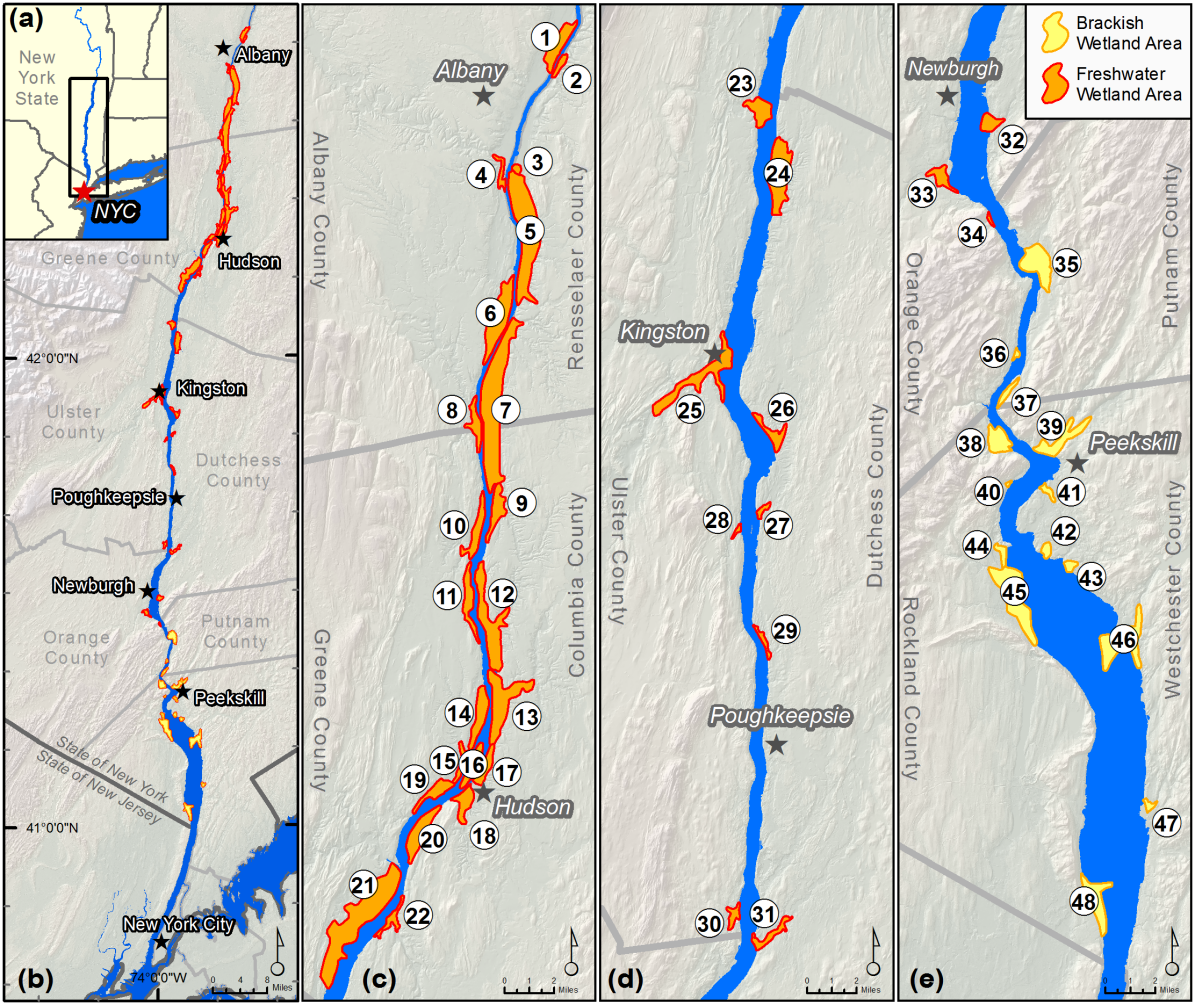
The HRE and its major tidal wetland systems. (a) Location of the HRE in New York State, northeastern USA; (b) overview of the tidal wetland systems of the HRE; (c)–(e) delineated tidal wetland systems in the HRE, from north to south. See S1 Table for a list of corresponding tidal wetland numbers and names.

The HRE, as defined by tidal influence, stretches approximately 245 km from the southern tip of Manhattan to the federal dam in Troy (in the States of New York and New Jersey, USA)(Fig 1b). In this stretch the river only descends approximately 1.5 meters, and unlike many other estuaries which flow through broad, low floodplains, its banks often feature steep topography, especially in its lower reaches [34]. These conditions result in the strong propagation of tides throughout the estuary and the distribution of approximately 2,800 hectares of tidal wetland in relatively narrow patches skirting the shores and islands in the river (Fig 1c–1e). The system is microtidal, with tide ranges approximately 1.3 meters near the river’s mouth decreasing to a meter or less around 100 km up river (near its deepest reach) and then gradually increasing to their maximum of ca. 1.7 meters in the uppermost reach of the estuary [35,36]. The extent of saltwater intrusion into the estuary and salinity levels fluctuate seasonally and with tides [28,37], with less than 20% of the tidal wetland area in the HRE occurring in places where the salinity levels are both high and consistent enough as to be classified as brackish wetlands [29]. The boundary between saltwater and freshwater also fluctuates between a defined salt wedge that forms during neap tides and low freshwater flows and an area of well-mixed fresh and salt water during spring tides and periods of high freshwater flow [28].

The habitats and biota of the HRE and its watershed are notably diverse [38]. The state recognizes 40 Significant Coastal Fish and Wildlife Habitat areas along the estuary—a designation afforded to particularly rich coastal habitats, and which is used to implement coastal policies. These designated tidal wetland complexes support a diverse fishery and provide critical habitat for two federally endangered species (a protective legal designation under the country’s Endangered Species Act), the Atlantic sturgeon (*Acipenser oxyrinchus*) and shortnose sturgeon (*A*. *brevirostrum*), as well as for myriad migrating and overwintering shorebirds and waterfowl [39]. Almost all of the estuary’s tidal plant communities have limited (<100) occurrences in the state and freshwater tidal swamps are considered the rarest among them [40]. These uncommon freshwater tidal communities are known to host numerous rare species of birds, odonates, fish, freshwater mussels, and plants.

In addition to its rich biodiversity, the Hudson River shore borders 78 municipalities, which to various degrees rely on the river as a source of commerce, a water supply, economic opportunity (including tourism) and recreation. Several large tidal wetland areas are likely buffering adjacent riverfront communities from storm impacts. With a long history of human habitation along the HRE, its tidal wetlands have been dramatically altered by river-bottom dredging and filling, bay impoundment by rail lines, nutrient and toxic chemical pollution, invasive species, and adjacent land use changes, among other factors [35,41]. For instance, ongoing dredging for improved navigation in the 56 most northern kilometers of the study area transformed a historically shallow and braided river channel into a deep main channel, resulting in the filling of over 1,700 ha (57%) of the historic shallow water and intertidal habitats [42]. Today, more than half of the tidal wetland area in the HRE is conserved, or protected to some degree from being altered for the purposes of development. Roughly 49% of the tidal wetlands occur in areas owned in fee by state agencies, municipalities, and non-profit organizations as parks or preserves; another 4% are owned by the United States military or New York State’s Office of General Services, or are on private properties with a conservation easement.

### Sea Level Affecting Marshes Model (SLAMM)

At the onset of this study, we selected SLAMM from a number of different computer programs available to simulate changes in tidal wetlands in response to SLR. SLAMM version 6.2 [33], and in particular its integrated accretion model and uncertainty analysis, presented a number of advantages in the context of our research needs. SLAMM has been used successfully to capture the general patterns of tidal wetland change as tested by hindcasts [20,27]. It has been applied in wetland systems in parts New York and Connecticut that are geographically adjacent to our research area, and thus have similarities in many physical and ecological respects. In addition, this program is freely accessible, benefits from a wide user base, and was well suited to the scale and data availability of our study area [32].

Any model for forecasting tidal wetland response to SLR has its strengths and weaknesses, and assumptions which must be considered in interpreting its projections. SLAMM associates different types of tidal wetlands to physical conditions and processes, such as elevation in the tidal frame (and inundation frequency), salinity, and accretion, and projects changes in land cover based on the projected changes in those conditions. A decision tree is used to determine the nature of land cover class changes, which are one-directional and take place in a step-wise fashion (i.e., in each time-step a habitat class can only change to the next class as defined by the decision tree, regardless of the magnitude of change in the conditions). This model’s framework is based on the assumption that inundation leads to the establishment of new tidal wetland, and does not account for the time it takes for wetlands to reach equilibrium based on a given level of inundation. The assumptions inherent to SLAMM and to our application of it to the HRE are further discussed in the results section as they pertain to their interpretation, as well as in the S1 Appendix.

We simulated changes in the estuary in 20-year intervals up to the year 2100, with high, medium, and low rates of sea level rise (HSLR, MSLR, LSLR, respectively) and high, medium, and low rates of accretion (HA, MA, LA, respectively). By comparing simulations with all of the different combinations of SLR accretion rates (nine in total) we were able to examine the model’s sensitivity to wide ranges of these two parameters, which have been shown to be the dominant and opposing drivers of change in tidal wetlands [14,43–45].

#### Spatial Data Sets

Input data sets to SLAMM included elevation, slope, and land cover, which originated from existing, publicly accessible data and were processed for our study using SLAMM and ArcGIS [46]. We calibrate a digital Elevation Model (DEM) derived from high resolution LiDAR [47] to a mean tide level of zero based on a modeled Hudson River vertical tidal datum [36,48], and then calculated the slope input dataset from the DEM [49]. The land cover dataset was created by combining a tidal wetland classification map of the Hudson River [50], the extent of the Hudson River [51], and non-tidal land cover classes from the National Land Cover Database [52]. Adjustments to the data sets were made to maximize accuracy, create a common 5 m^2^ resolution, and to simulate a common starting time frame based on the tidal wetland map of 2007. We further processed the land cover dataset by using a “Time Zero” SLAMM simulation, which applies the model with no change in sea level to the existing conditions, and used the resulting land cover data as the input into all of the subsequent SLR and accretion scenario simulations. The methodology for the processing of SLAMM input data is further detailed in the S1 Appendix.

**Fig 2:**
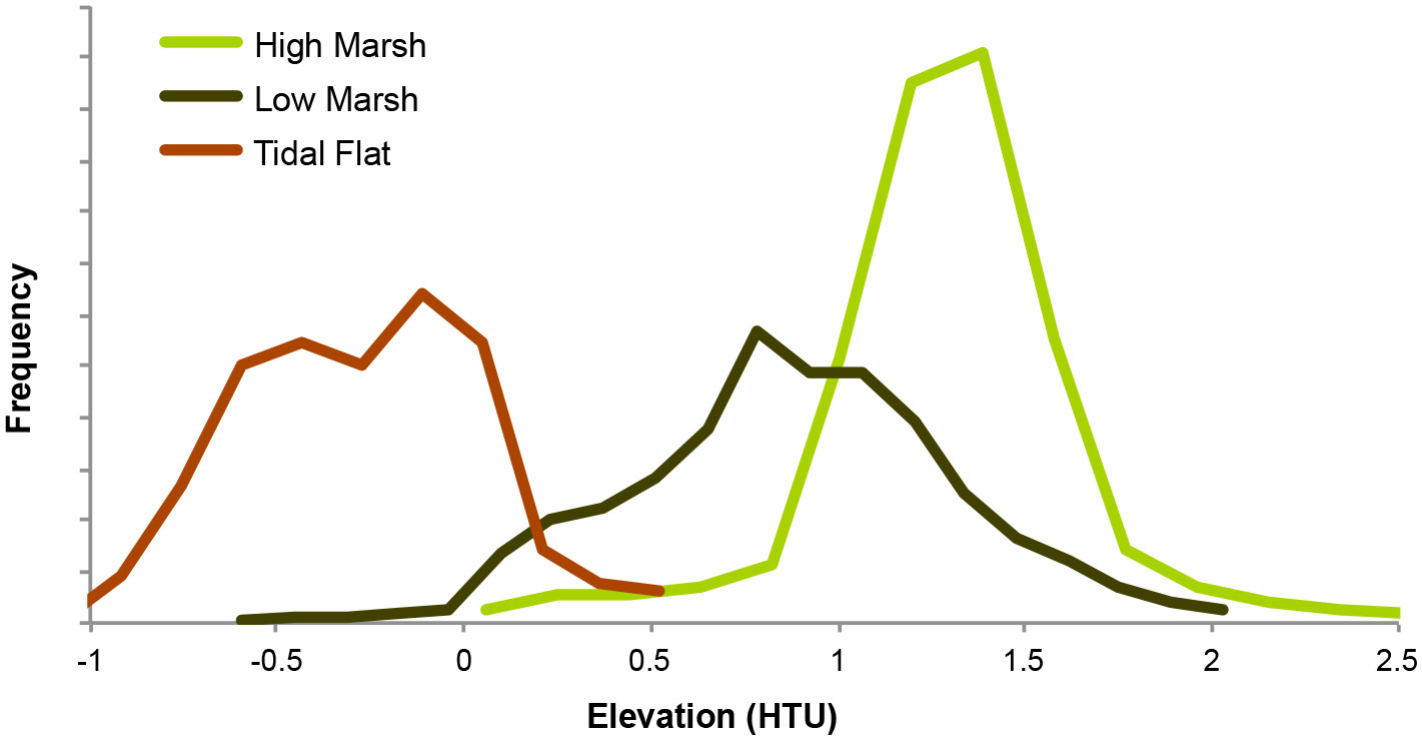
The frequency of occurrence of the model’s tidal wetland classes along an elevation gradient. Data shown are for the northern portion of the study area, and exclude any values that were well outside of the elevation range of each wetland class. This pattern of elevational frequency for the three tidal wetland classes is representative of both the north and south sections of the study area.

An elevation analysis of the mapped wetland data revealed that several categories were largely overlapping, and some categories (e.g., tidal swamp) did not align well with the SLAMM conceptual model in terms of their elevation in the tidal frame. This misalignment was due in part to a classification scheme in the original wetland mapping that was not intended for use in SLAMM, and in part likely due to the freshwater conditions dominating the HRE [2,30](S1 Appendix). Since SLAMM version 6.2 has a pre-defined class-switching decision tree that is, for the most part, not customizable, we used the information from the elevation analysis to combine our wetland classes into a simplified classification with three classes that transition sequentially with increasing inundation: High Marsh, Low Marsh, and Tidal Flat (Fig 2, Table 1, S1C Fig). This re-classification is the key aspect of our adaptation of SLAMM to freshwater tidal conditions, as it uses only classes with simple transition rules that are not dependent on the influence of salinity, yet adequately reflects the zonation of both the brackish and freshwater wetlands of the HRE. We raised the default minimum elevation of the high marsh category from 0.5 to 0.8 Half Tide Units (HTU)(half of the elevation of the full range of tide, spanning from Mean Lower Low Water and Mean Higher High Water) to better reflect the observed data and to differentiate between the high and low marsh types.

**Table 1:** Re-classification of the 2007 mapped wetland classes into SLAMM classes with model elevation ranges. Elevation ranges are in Half Tide Units—the difference between Mean Higher High Water or Mean Lower Low Water and Mean Tide Level.

Land Cover Class (SLAMM category name)	Elevation Range (HTU)	2007 Mapped Wetland Classes
Developed Upland, Undeveloped Upland	> 1.5	Upland
High Marsh (Irregularly Flooded Marsh)	0.8–1.5	Wooded Swamp, Scrub/Shrub, *Phragmites australis*, Salt Meadow
Low Marsh (Regularly Flooded Marsh)	0–1.2	*Typha angustifolia*, Upper Intertidal, *Spartina alterniflora*
Tidal Flat	-1–0	Vegetated Lower Intertidal, Unvegetated Flat
Estuarine Open Water	< -1	Submerged Aquatic Vegetation, *Trapa natans*, Open Water

#### Sea Level Rise Projections

Rates of relative SLR in the northeastern United States coastal zone generally exceed eustatic rates, with coastal zones around New York City experiencing SLR at approximately twice the global rate due to local land subsidence and changes in Atlantic Basin currents [53,54]. In the Hudson River, SLR rate projections inclusive of the highest values fall in the range of 28 to 190 cm in the 21^st^ century [53,55,56]. Observed rates during the years 2000–2014 (based on tide gauge readings at the Battery in Manhattan [54]) are approximately 0.7 cm/year, which falls within the middle range (25^th^–75^th^ percentile) of local projections.

We used New York State ClimAID local SLR projections as a guide for our three SLR levels, including projections for “Region 4 –New York City” and “Region 5 –Troy Dam”—the southern and northern portions of our study area, respectively [56]. Slightly higher local rates of SLR are projected for areas south of the City of Kingston (ca. 145 km up the estuary) due to more pronounced vertical land movement (resulting from glacial isostatic adjustment) in this area [55]. We used the low and high estimates of the ClimAID projections (10^th^ and 90^th^ percentiles, respectively) as our low and high SLR inputs, and the mean of the middle range (25–75^th^ percentile) as our medium SLR input (Table 2).

**Table 2:** SLR projections in centimeters (rounded to the nearest whole number) used in SLAMM simulations for the north and south sections of the HRE.

Location	Rate of SLR	Year of Simulation				
		2020	2040	2060	2080	2100
**South**	**Low**	3	9	17	25	35
	**Medium**	7	22	42	65	88
	**High**	14	47	89	137	187
**North**	**Low**	2	6	12	18	25
	**Medium**	6	20	37	57	78
	**High**	13	45	85	130	177

#### Rate of Accretion

The building up of the tidal platform by the process of accretion, including mineral and organic contributions, allows tidal wetlands to adapt to changes in sea level through feedbacks that promote vertical changes in the wetland platform [14,43,44,57]. The HRE has a complex and dynamic sedimentary regime [58,59], which combines with likely high organic matter contributions to overall accretion [60]. Estimates of accretion or sedimentation rates from various studies on HRE wetlands (including the subtidal shallows) vary widely, ranging approximately 0.3–29 mm/yr depending on time scale, sampling method, wetland location in the estuary and wetland type [29,61–63]. Empirical data or model estimates that combine levels of available sediment, deposition rates and organic inputs, and relate them to long term rates of accretion were not available on an estuary-wide basis.

**Fig 3:**
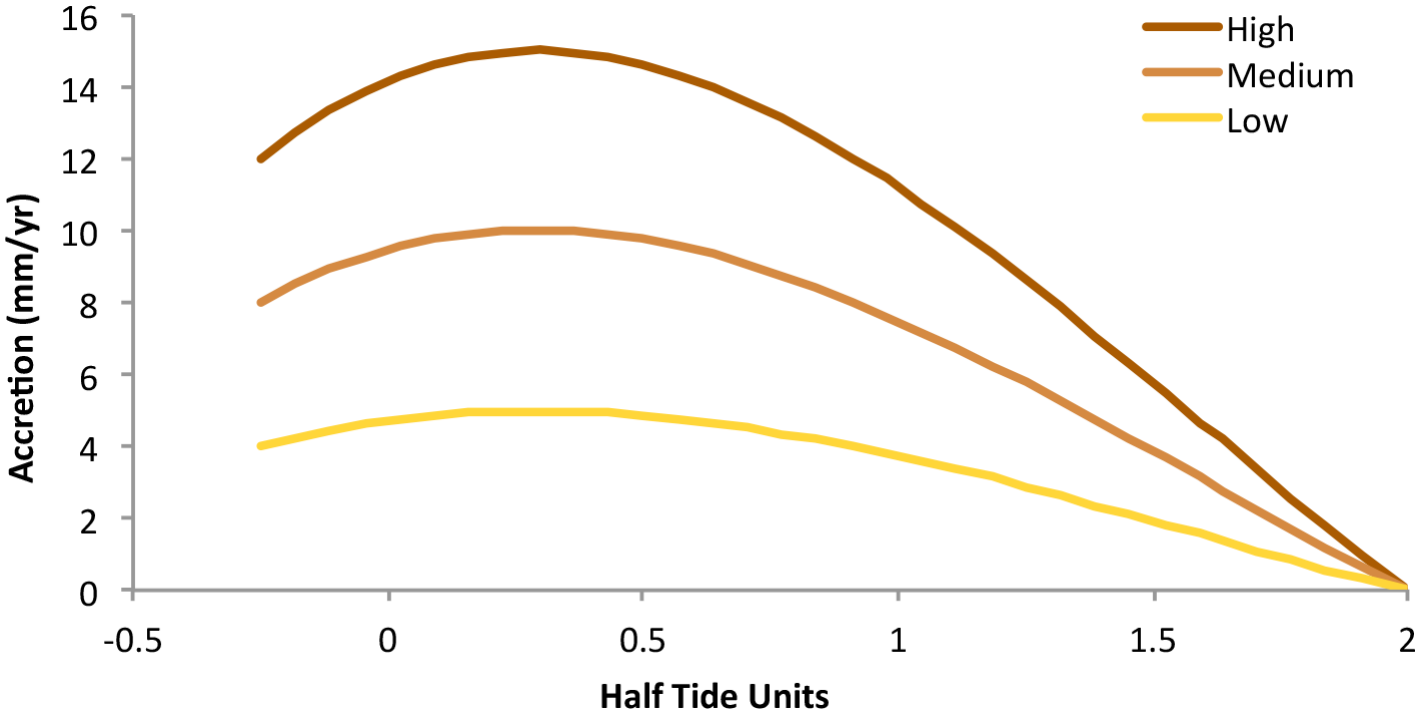
The three generic accretion curves used to parameterize high, medium and low marsh accretion rates in SLAMM models. Elevation is in Half Tide Units [HTU]—the difference between Mean Higher High Water or Mean Lower Low Water and Mean Tide Level.

To approximate accretion rates across elevation and tidal wetland types, we used three generic curves that mechanistically describe the feedbacks between marsh elevation and accretion [14,33,43]. We defined the curves using three maximum values that capture the likely range of possible maximum accretion values over the study’s timeframe (Fig 3), and used these curves to derive accretion rate parameters for high and low marsh (based on their elevation range in the model)(S1 Appendix). In tidal flats we applied a constant rate of half the maximum accretion rate [18]. We used this rough estimate—a simplification of the more nuanced conditions that have been shown to exist in some freshwater tidal flats [14], and which could vary particularly depending on the vegetated or non-vegetated nature of the flat—due to a lack of data in our study area. Our use of a range of accretion scenarios represents an intentional effort to produce results that will include a wide range of possibilities, in lieu of the ability to parameterize more accurate accretion models across the estuary with the currently available information.

### Data Analysis

We examined model results in terms of 1) projected tidal wetland extent and composition, 2) wetland resilience and migration, 3) the geography of projected changes in the HRE, 4) model sensitivity and 5) model uncertainty. Wetland resilience measures include loss, change, and persistence of existing wetlands and the formation of new tidal wetland in former upland areas (i.e., wetland migration). To quantify resilience, we used ArcGIS to compare the raster of initial conditions with the raster file results of different scenarios (S1 Appendix). In the context of wetland resilience and migration, we also sought to evaluate the contribution of currently conserved lands to the future wetland area, and to this end we used ArcGIS to relate a raster of conserved lands (current as of April 2015) to the SLAMM results. Additionally, we aimed to quantify the future conflict between marsh migration and developed lands (i.e., those areas where land uses would prevent marsh migration, such as buildings and roads). Our SLAMM simulations intentionally allowed developed areas to convert to tidal wetlands (i.e., developed areas were not “protected” from change). We then post-processed the simulations’ raster outputs in order to report on tidal wetland projections in the estuary with the exclusion of developed uplands, while also quantifying the level of potential conflict between future wetlands and currently developed areas (S1 Appendix).

To analyze the impact of geography on model results, we delineated the major tidal wetland systems along the Hudson (Fig 1c–1e, S1 Table). This delineation was based on New York State Department of Environmental Conservation’s named tidal wetland systems and the state’s Department of State designated Significant Coastal Fish & Wildlife Habitat areas [39], with two additions based on the input data and projections. In all, we delineated 48 wetland areas, totaling nearly 2,700 hectares (almost 95% of the total wetland area in the estuary). We used ArcGIS to analyze the projected wetland changes under different SLR and accretion scenarios by wetland system. This allowed us to examine which wetland systems are most likely to undergo change or exhibit resilience, in terms of wetland loss, persistence or expansion. We also use these wetland areas as the basis for uncertainty analyses for site-specific conservation planning purposes.

### Uncertainty Analysis

Uncertainty analysis is important for interpreting SLAMM model results given the errors inherent in the model’s inputs [23,45], but was prohibitive at the scale of our entire study area (which spans an area with two different sets of SLR projections). The SLAMM 6.2 model provides a built-in uncertainty analysis function employing a Monte Carlo method in which model parameter values are randomly selected from user-defined probability distributions on a multiplier scale, for a specified number of model iterations [33]. Examining the full range of results and the level of consensus among the results of these iterations provides information about the range of possible changes and the level of certainty in the projected changes. We ran uncertainty analyses on individual wetland system sites, varying the rate of SLR, maximum rates of accretion, the DEM and the tide range from the MSLR-LA scenario based on estimated probability distributions and known error in these parameters (S1 Appendix).

We created a python script geoprocessing tool to visualize the results of the uncertainty analyses. The tool aggregates the results of all iterations and outputs a raster of the wetland class projected in the majority of iterations for each pixel, along with a classification of the percent of models that projected that class (e.g., <50%, 50–90%, >90%). Following the example of Clough et al. 2014 [23] we also developed a tool that quantifies the likelihood of specific, user-specified changes in class from the initial condition to a specified time frame, which allows us to generate percent likelihood maps for various predicted future conditions (e.g., the percent likelihood of the conversion of any land cover type to open water by 2100, the percent likelihood of tidal wetland resilience by 2060). In the results section we present an example of the uncertainty analysis for the Iona Island Marsh tidal wetland area (Fig 1e, #38).

## Results and Discussion

Based on current observed rate of SLR and reported rates of accretion, the medium SLR and low accretion scenario (MSLR-LA) most closely correlates with the observed conditions for both sections of the Hudson, and thus is referred to as the “current trend” scenario [54,58,62,64,65]. Results of high SLR simulations represent more dramatic yet plausible changes, which may result from more drastic increases in rate of SLR due to the accelerated melting of the West Antarctic and Greenland ice sheets [55,66]. Since current observed SLR rates already well exceed those of the low SLR value, the results of the LSLR scenarios were used only to examine model parameter sensitivity, and are not otherwise discussed in the this section. We also focus on the results for the full time span of our study; all of the figures and trends described below are based on the projections for year 2100, except where otherwise noted. In some instances we also report the results of the “consensus” of the MSLR and HSLR scenarios—this is the area where all six simulations overlapped in their projection of the condition reported (e.g., all scenarios projected some type of tidal wetland by year 2100).

### Projected Tidal Wetland Extent and Composition

Based on the “Time Zero” simulation the Hudson River supported approximately 2,800 hectares of tidal wetlands in the baseline year 2007. All MSLR and HSLR modeled scenarios projected a net increase in total tidal wetland area (including high marsh, low marsh, and tidal flat) through the century due to the upland migration of marshes, ranging from 465–1,600 hectares. Projections of total wetland extent were lowest in the HSLR-LA scenario (3,300 ha) and reached a maximum of 4,400 hectares in the HSLR-HA scenario. The wetland area of consensus among the six scenarios totaled 2,260 hectares.

**Fig 4:**
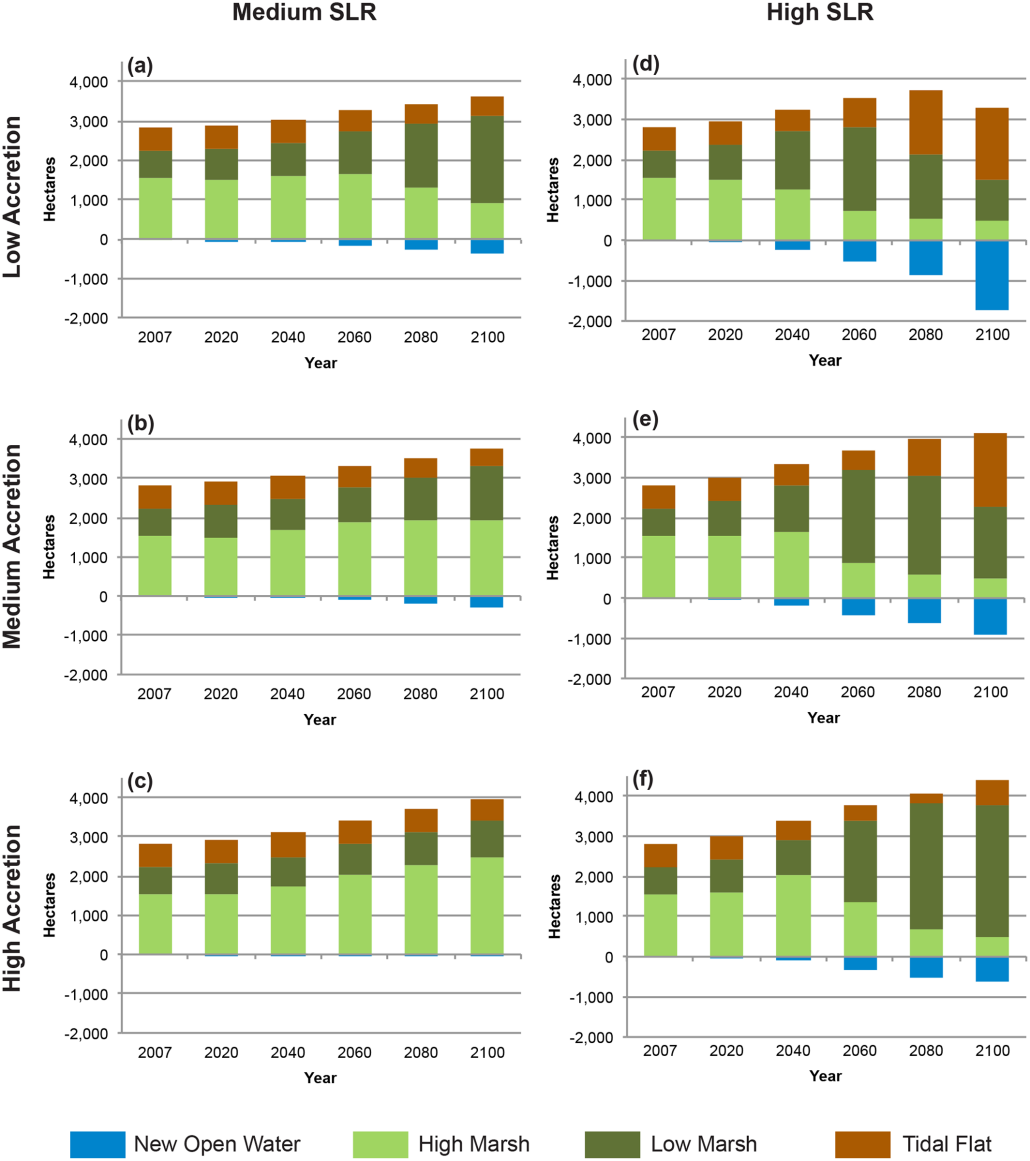
Change in wetland extent and wetland classes. (a) MSL-LA, (b) MSLR-MA, (c) MSLR-HA, (d) HSLR-LA, (e) HSLR-MA, and (f) HSLR-HA scenarios.

In all MSLR and HSLR scenarios, total wetland extent increases driven by new wetland formation were partially offset by the transition of existing wetlands to open water. The HSLR scenarios projected the greatest losses of existing wetland areas to permanent inundation by the end of the century, ranging from ca. 600–1,700 ha (Fig 4d–4f). In the HSLR-LA scenario, gains in new wetlands only exceeded the extent of losses by 22%.

Our findings suggest that in the HRE, overall loss of tidal wetland area to inundation in this century could be offset if marsh migration into upland areas is realized. This may be in contrast to the some saltwater coastal zones in southeastern New York State, where a rapid loss of tidal wetland has been documented [67–69]. This difference could be the result of several factors or their combination, including a differing adaptive capacity of salt and freshwater systems, higher rates of local SLR due to subsidence, or the generally higher density of development that is associated with southeastern New York’s saltwater coastal zones, which limits wetland migration pathways [21,24,53,70].

The composition of tidal wetland habitats is projected to shift considerably in the current trend and all high SLR scenarios (Fig 4a and 4d–4f, Table 3). Under these scenarios, high marsh area will decrease and low marsh will increase over the century. These same four scenarios will also see the most significant increases in estuarine open water (i.e., permanent inundation of wetland and upland areas), and all HSLR scenarios also project increases in tidal flat. These trends result from a rate of SLR that exceeds the capacity of the high marsh to accrete. In comparison, the MSLR-MA and MSLR-HA scenarios both project increases in high marsh and low marsh, with only slight losses of tidal flat (Fig 4b and 4c). With these more moderate rates of SLR, a moderate or high rate of accretion allows for low and high marsh to maintain their relative elevation in the tidal frame.

**Table 3:** Wetland change (ha and %) by time step in the MSLR-LA and HSLR-LA scenarios. Numbers in each time step represent changes from the previous time step (with 2020 representing the change from the Time Zero simulation of year 2007).

		2020		2040		2060		2080		2100		Total by 2100	
		ha	%	ha	%	ha	%	ha	%	ha	%	ha	%
**Medium SLR**	**Total Tidal Wetland**	75	3%	154	5%	214	7%	167	5%	204	6%	814	29%
	**High Marsh**	-58	-4%	137	9%	45	3%	-366	-22%	-390	-30%	-632	-41%
	**Low Marsh**	121	17%	17	2%	217	26%	593	56%	593	36%	1542	220%
	**Tidal Flat**	12	2%	0	0%	-48	-8%	-60	-11%	1	0%	-96	-17%
	**Open Water**	11	0%	44	0%	97	0%	132	0%	103	0%	388	1%
**High SLR**	**Total Tidal Wetland**	159	6%	290	10%	289	9%	187	5%	-459	-12%	466	17%
	**High Marsh**	-34	-2%	-261	-17%	-527	-43%	-151	-21%	-94	-17%	-1067	-69%
	**Low Marsh**	187	27%	585	66%	609	41%	-515	-25%	-544	-35%	322	46%
	**Tidal Flat**	6	1%	-35	-6%	207	38%	853	112%	179	11%	1210	209%
	**Open Water**	41	0%	212	1%	272	1%	326	1%	901	3%	1752	6%

The current extent of low marsh was sustained or increased in all MSLR and HSLR scenarios, in part as a consequence of the conversion of existing high marsh to low marsh. However, in the HSLR-LA and HSL-MA scenarios, the initial gains projected for low marshes decline in later time frames (2080 and 2100)(Fig 4d and 4e). In our model, the fate of tidal flat habitats strongly reflects rate of SLR, with MSLR leading to slight losses and HSLR leading to potentially great gains (reaching over 300% in the low and medium accretion scenarios), mostly at the expense of wetlands higher in the tidal frame (Fig 4). Initial transitions between high and low marsh may be slightly exaggerated in our model, due to the upward adjustment of the minimum elevation value for high marsh, and the elevational overlap between the high and low marsh types. The long-term patterns, and particularly those of transitions from low marsh and tidal flat, are little or not at all affected by this model adjustment. Projected wetland losses (the transition from tidal flat) are driven in part by our static estimated rate of accretion for this wetland type, and can be improved when better data are available.

**Fig 5:**
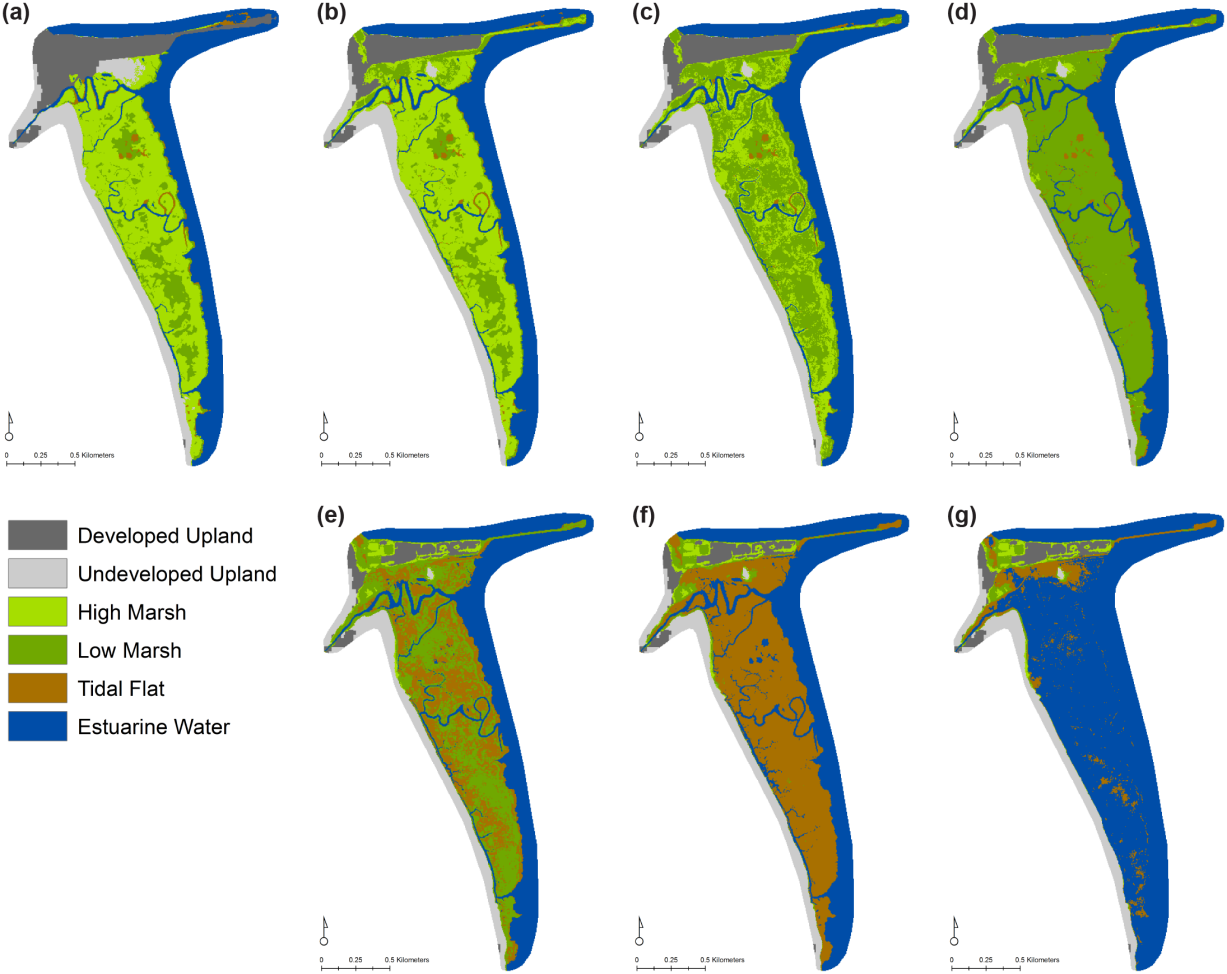
Projections for the Piermont Marsh tidal wetland area (Fig 1c, #48). (a) Time Zero (current conditions); MSLR with (b) HA, (c) MA, and (d) LA by year 2100; HSLR with (e) HA, (f) MA, and (g) LA by year 2100.

Two recent studies applying SLAMM to the coasts of New York and Connecticut projected similar patterns of composition shifts, with high marsh conversion to low marsh increasing with higher SLR rates, and the highest rates of SLR also driving conversion of low marsh to tidal flats [23,71]. The New York study by Clough et al. [23] overlapped in extent with ours at Piermont Marsh; this is a 109-hectare tidal wetland system dominated by high marsh that is located at the southern end of the HRE, and is the most saline of the estuary’s brackish wetlands (Fig 1c, #48). The results of the two studies are difficult to compare directly due to differences in model parameters (e.g., rates of SLR simulated, accretion model, wetland classes), which result in part from model parameterization that is based on relatively large study areas which are mostly non-overlapping, and in part from study design (e.g., we tested a range of accretion scenarios, none of which correspond exactly with the model used by Clough et al.). Our study’s results from the scenario with the largest projected changes (HSLR-LA, Fig 5g) were more drastic than any projections by Clough et al., in large part due to our comparatively low rate of accretion in this scenario. However both studies found a similar pattern, with high marsh exhibiting resilience to change in simulations with low rates of SLR and/or in short time frames, but ultimate conversions to low marsh and tidal flat with higher rates of SLR by 2100. Comparable patterns have also been projected by a recent study in the San Francisco Bay Estuary that similarly compared a range of SLR and accretion rate scenarios using the Marsh Equilibrium Model [24].

### Wetland Resilience and Migration

To assess tidal wetland resilience, we classified land cover conversions into the following categories: most resilient (wetlands that persist in their class), somewhat resilient (wetlands that change in class), new wetlands (wetlands forming in upland areas), and lost wetlands (those converted to open water). While newly formed wetlands are not a measure of the ability of a wetland to persist, they contribute to the overall resilience of the tidal wetland system over time.

**Fig 6:**
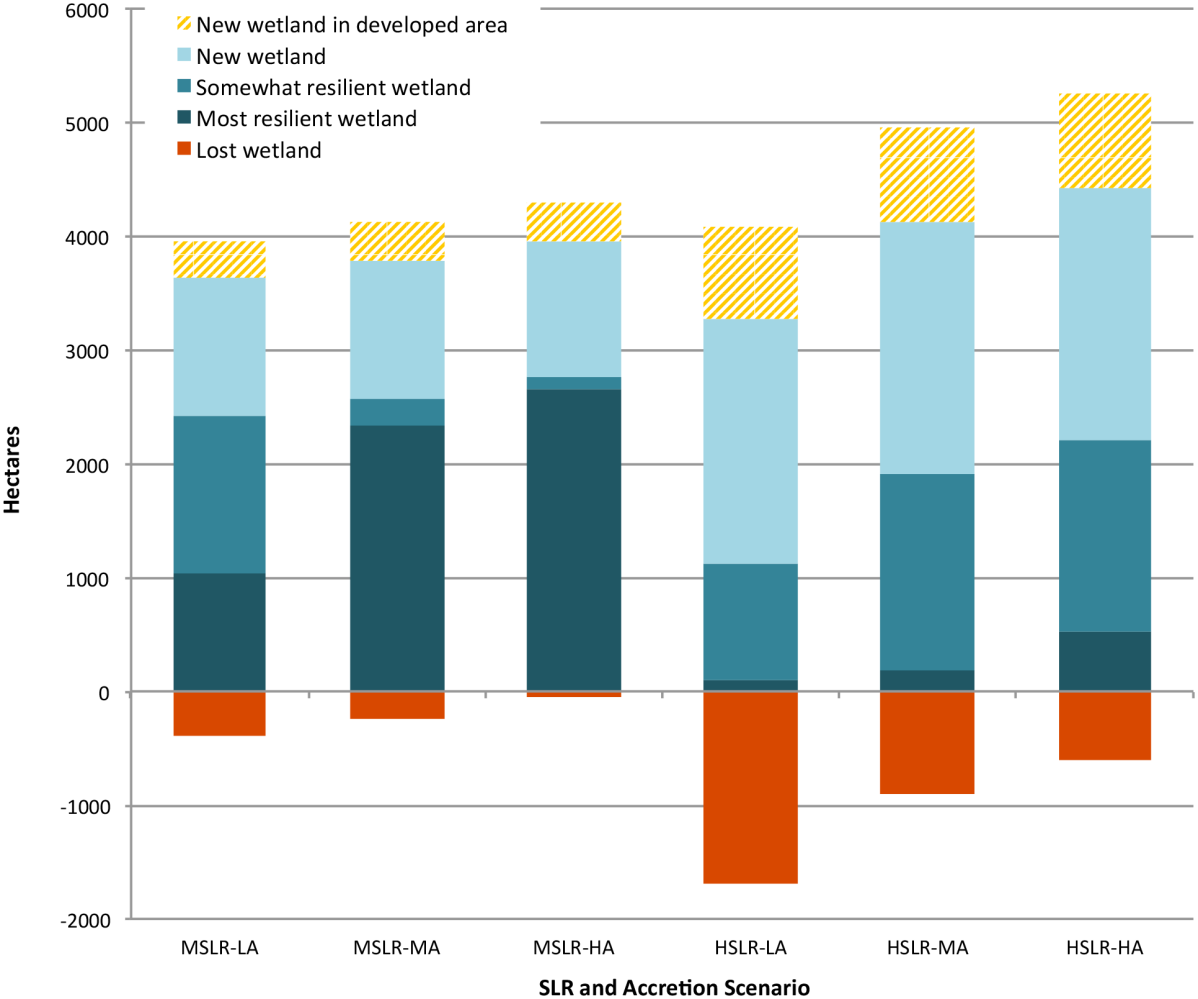
Projected wetland resilience and loss in HRE wetlands by year 2100. Somewhat resilient wetlands are those that experience a change in class, while most resilient wetlands are those that maintain the same class through the century.

The consensus area of projected wetland provides some insight into the likely envelope of future tidal wetlands based on the two driving parameters of SLR and accretion. Across the estuary this area of overlap in projections measured ca. 99 ha of most resilient wetlands, 1,020 ha of somewhat resilient wetlands, 1,140 ha of new wetland, in addition to ca. 60 ha of lost wetland. Under the current trend scenario, 49% of current tidal wetlands are projected to undergo a change in class (e.g., high marsh to low marsh), 37% are projected to be resilient enough to adapt to SLR without class conversion, and 14% of existing tidal wetlands are projected to become permanently inundated. In this MSLR-LA scenario the biggest projected change in any land cover class (including tidal wetlands and uplands) is the conversion of over 1,100 hectares of high marsh to low marsh. The HSLR-LA simulation indicates a more extreme outcome in terms of overall tidal wetland area and resilience by the end of the century; in this scenario the most resilient wetlands represent only 4% of the total and 60% of tidal wetlands in 2007 are lost to inundation (Fig 6).

Under the current trend scenario, SLAMM projected ca. 1,200 hectares of new wetland forming in undeveloped uplands. These migrated marshes represent 33% of the total projected tidal wetland area. Of the wetlands projected to migrate into undeveloped upland areas, approximately 47% would occur in already conserved areas, and the remaining 53% would occur in places that are not conserved in fee or by easement, and which are more susceptible to future development. In the HSLR-LA scenario the relative importance of new wetlands is much greater, with ca. 2,150 ha of new wetland representing 66% of the total wetland area. The conflict of migrating marshes with developed areas is also expected to be exacerbated by high rates of SLR, measuring over 800 ha in all three HSLR scenarios as compared with 330 ha under the current trend scenario (Fig 6). These projections highlight the need for changes to regulatory conservation of tidal wetlands to accommodate dynamic wetland boundaries [72], the importance that acquisition and other conservation measures have in protecting future tidal wetlands and the need for adaptive planning in developed coastal areas.

We consider existing wetlands that are resilient to SLR over time to be a high priority for conservation, as they represent places that provide important enduring ecosystem services and species habitat, and will likely serve a critical bridging function for new wetlands. New wetlands, formed by the migration of high marsh into upland areas, are a key component of long-term marsh resilience, and become increasingly important components of the tidal wetland portfolio under scenarios with higher rates of SLR. The availability of adjacent undeveloped upland areas varies between wetland sites, and those with constrained migration pathways are more vulnerable (i.e., less resilient) to shifts in habitat composition and inundation [24]. Barring saltwater intrusion, freshwater tidal wetlands may generally be more resilient to SLR than salt or brackish marshes [21,73], and thus there may be variation in resilience in the tidal wetlands of the HRE such that the currently brackish wetlands and those freshwater ones closest to them (i.e., those that will become brackish in the case of more pronounced saltwater intrusion into the estuary) may be more vulnerable to future shifts. Variation in the position of wetlands systems relative to the river (e.g., in coves, around islands), shoreline topographies, and adjacent land uses will also likely impact wetland resilience to SLR.

### Geography of Change and Resilience

**Fig 7:**
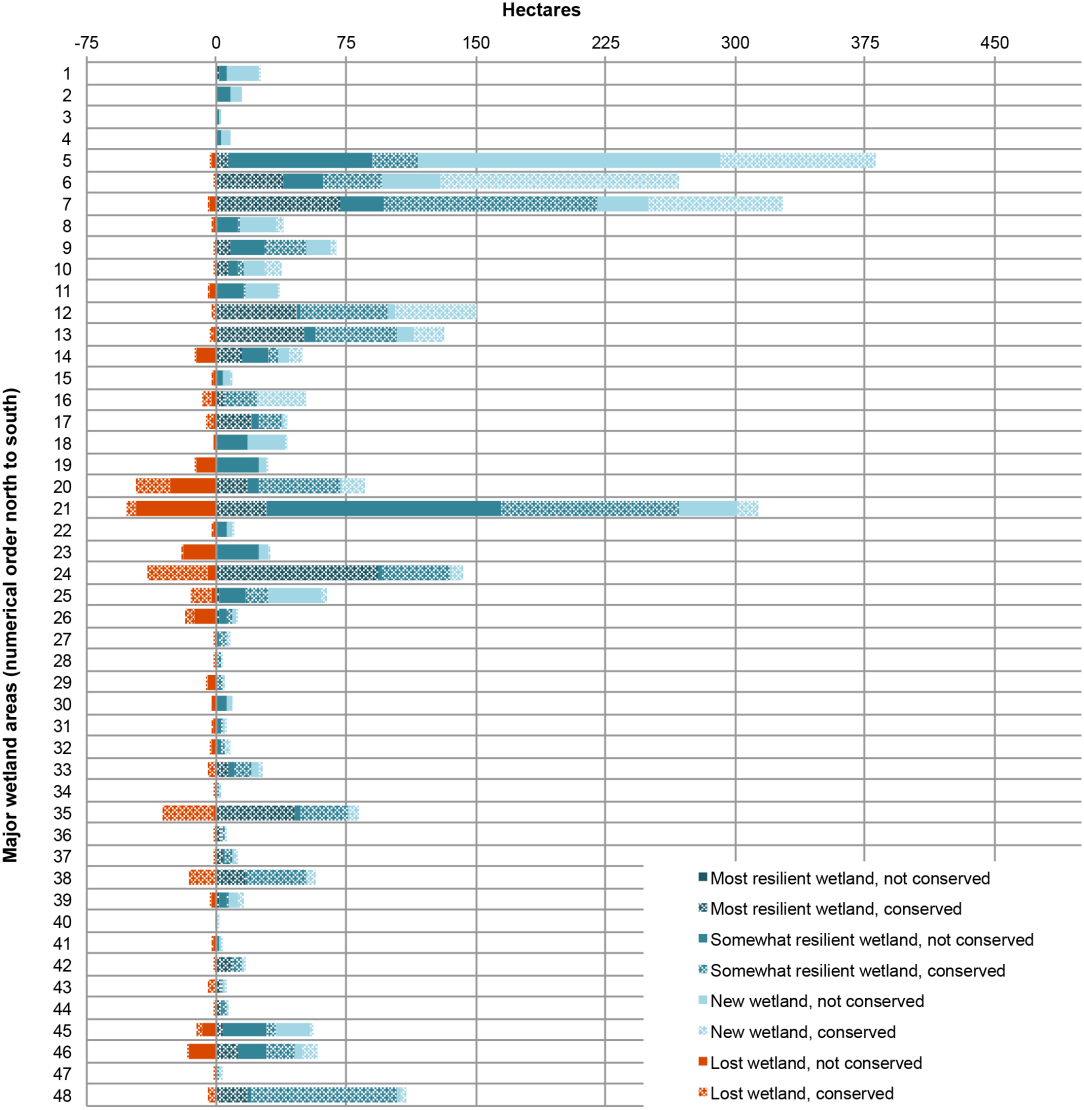
Wetland resilience and loss projected within the wetland systems of the HRE under the current trend scenario. Somewhat resilient wetlands are those that experience a change in class, while most resilient wetlands are those that maintain the same class through the century. Fig 1c–1e shows the location of the tidal wetland systems in the estuary, and the S1 Table relates wetland system numbers to names.

The HRE’s major tidal wetland systems, which encompass approximately 95% of the total wetland area in the estuary, are projected to vary in their response to increases in SLR. The northern reach, which is characterized by generally larger tide ranges and wider floodplains, hosts the largest wetland complexes (Fig 1b, S1 Table) and is projected to experience the largest tidal wetland expansions in the coming century. Based on the MSLR-LA scenario, only three of these northern wetlands combine to support over 50% of all projected new wetlands in the estuary (Figs 7 and 1c #5–7). At Papscanee and Campbell Islands (wetland #5) the wetland expansion area on private, non-conserved lands measures ca. 175 ha—by far the largest such expansion in the estuary, representing a notable conservation opportunity. Wetlands with the greatest projected losses in the current trend scenario also tended to gain relatively little new wetland area, due at least in part to steep shoreline topographies (e.g., Fig 7 #20, 21, 24 and 35).

Wetland resilience also varied across the estuary. Locations with the highest projected percentage of most resilient wetland in the MSLR-LA scenario are characterized by currently having relatively high proportions of low marsh (and in particular cattail-dominated marsh)(e.g., Fig 7 #17 and 24). The distribution of low marsh in our model is skewed toward the higher elevations in its range (i.e., its abundance peaks around 0.8 HTU, in an elevational range of 0–1.2 HTU), and thus it can tolerate relatively larger increases in sea levels before the model projects a conversion to tidal flat. Seven wetland areas in the estuary are considered to exhibit the highest overall resilience, with currently existing wetland areas accounting for ≥90% of the total wetland area projected by the end of the century (including new wetland)(Fig 7 #17, 19, 24, 35, 37, 38, and 48). Marsh migration potential (as described in a previous section) and overall wetland size can also be considered important components of tidal wetland resilience. The high productivity of freshwater tidal wetlands may provide an important boost to accretion and overall adaptability as compared with saline coastal wetlands [24,70], which would add a geographic gradient to wetland resilience in the HRE that is not accounted for by our study.

### Model Sensitivity

**Fig 8:**
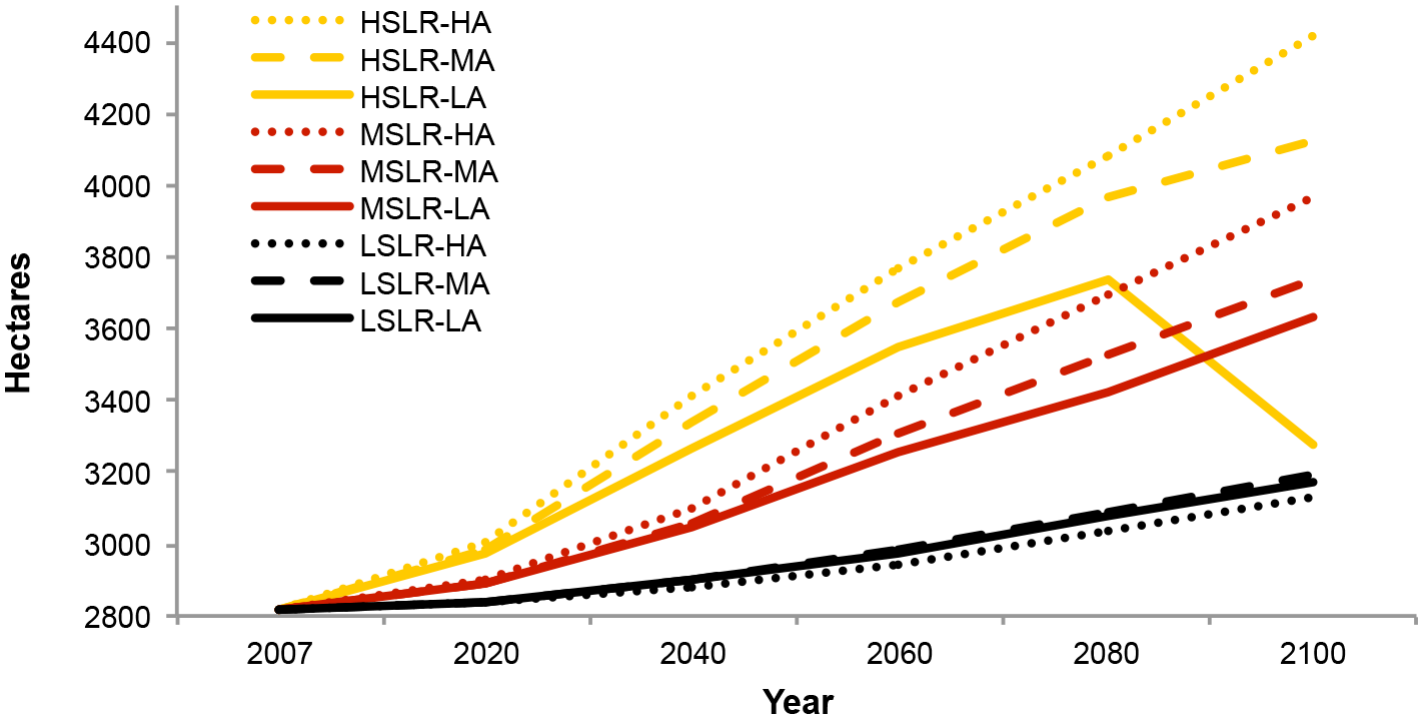
Total tidal wetland area projected by the nine SLR and accretion scenarios. These results include high marsh, low marsh and tidal flat, and exclude developed upland areas that were projected to convert to wetland.

Projections of total wetland extent and migration were primarily driven by the SLR parameter, with rate of accretion acting as a secondary driver (Fig 8). For instance, the MSLR-LA scenario projected ca. 1,200 hectares of new wetland while the HSLR-LA scenario projected ca. 2,200 hectares of new wetland—a difference of 1,000 hectares (Fig 6). In contrast, varying the accretion level between low and high in a medium SLR scenario resulted in a difference of only 4 hectares of projected new wetland. The impact of differing rates of accretion on total wetland extent and composition was greatest under HSLR scenarios and in time frames of 2060 or later (Figs 4 and 8). Accretion also had a pronounced effect on resilience measures, with increased accretion rates boosting the proportion of most resilient wetlands to somewhat resilient wetlands as well as decreasing wetland losses within each SLR level (Fig 6, S4 Table).

The sensitivity of a model comparing different rates of SLR and accretion to each of these factors necessarily depends on the ranges of the parameters tested. In our study we aimed to test a wide range of SLR and accretion rates, and the differences in projected SLR for the HRE affected the measures of wetland extent and migration more dramatically than the estimated possible range of accretion. This highlights a need for updated or refined eustatic and relative SLR projections in order to produce accurate tidal wetland models. In model simulations with custom rates of SLR by year 2100, such as in our study, SLAMM scales the rate to the A1B maximum scenario described by the Intergovernmental Panel on Climate Change [33,74]; this is another element of SLR projections that may be refined with new data and models. Our results also indicate that a better understanding of local accretion rates is important for anticipating the resilience of tidal wetlands in the HRE. New empirical data for accretion rates across the estuary’s extent and wetland elevation gradients will allow for refinements of the generalized accretion model used in our study and also for projecting trends at a finer scale. Inclusion of both sediment availability and organic inputs into accretion measures may be especially important in our study area due to the high relative impact of organic inputs in found in some lower salinity systems [24,44]. The overall patterns of model sensitivity found by our study mirror those of other studies where sensitivity to both rates of SLR and accretion were tested [24,45].

### Uncertainty Analysis: Iona Island Marsh

**Fig 9:**
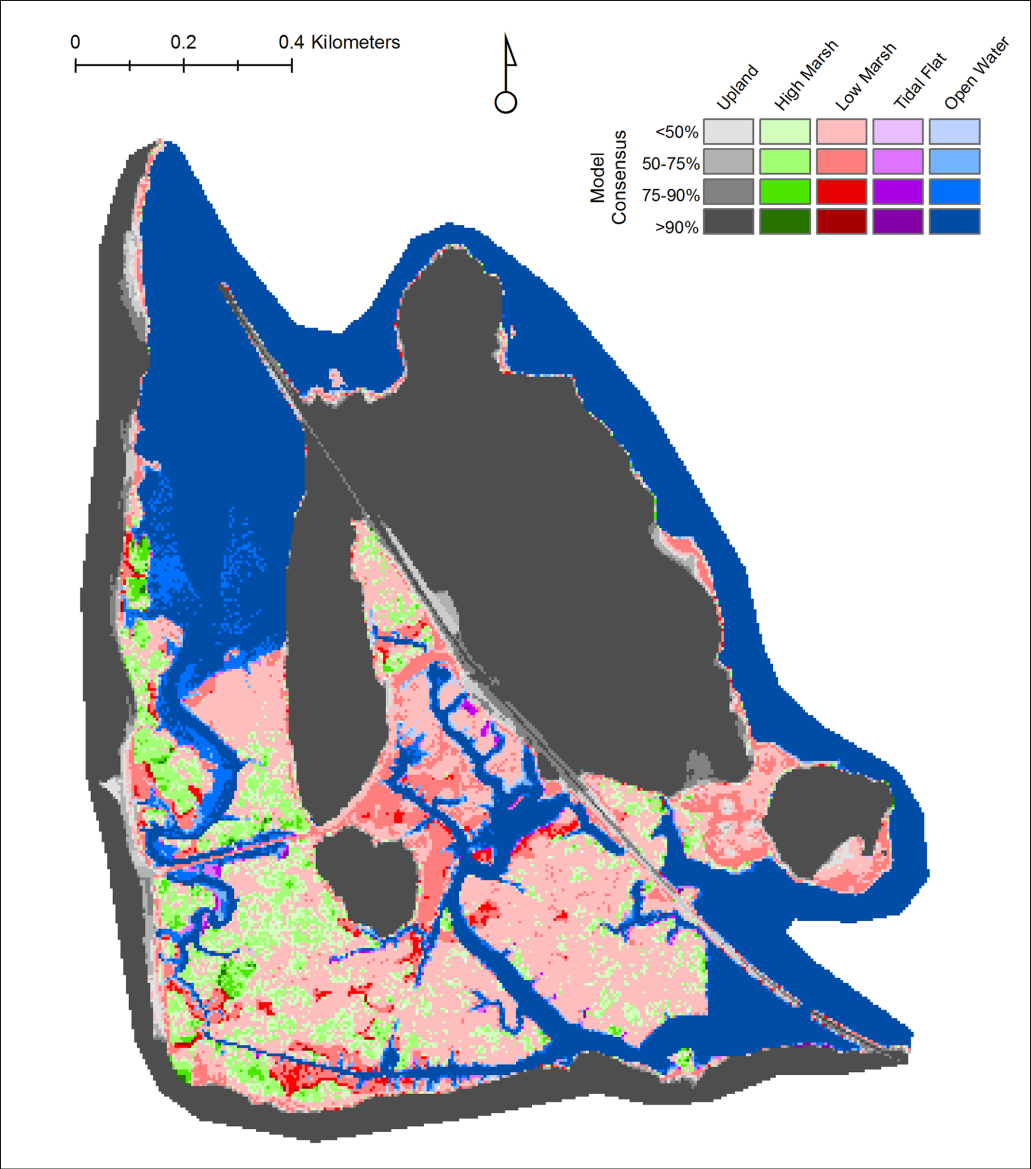
Majority projections of uncertainty analysis iterations for Iona Island Marsh by year 2100.

Iona Island Marsh is a brackish wetland located in the southern portion of the HRE (Town of Stony Point, Rockland County, NY; Fig 1e #38) that is managed as part of the Hudson River National Estuarine Research Reserve. We used the uncertainty analysis, with the geoprocessing tools described in the Materials and Methods section and probability distributions for SLR by 2100, the DEM, accretion maxima, and greater diurnal tide range (S1 Appendix) to produce outputs that account for the known uncertainties in these model inputs. The results showed a wide range of projected changes in tidal wetland composition, but a pattern of high marsh conversion to low marsh over the century was dominant, as well as an increase in estuarine open water (i.e., wetland inundation)(Fig 9). Examination of the majority class map for Iona Island Marsh by year 2100 indicated that the uncertainty analysis iterations were most consistent (i.e., highest percent values) in projecting inundation of tidal flat habitats, and in small areas where high or low marsh are likely to be resilient throughout the century.

**Fig 10:**
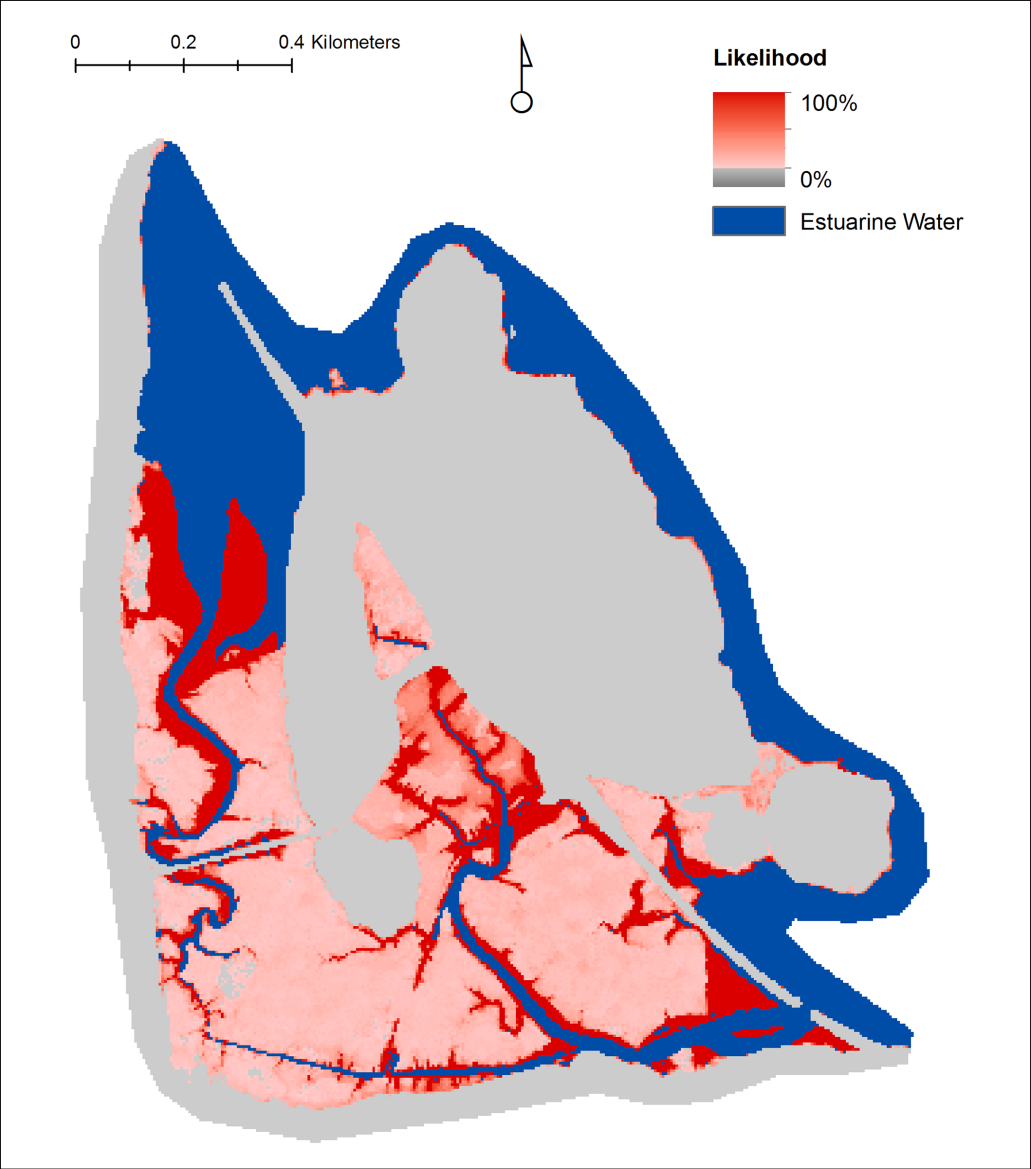
Percent likelihood of the inundation of any type of exiting tidal wetland by the year 2100 at Iona Island Marsh.

Since this entire wetland system is owned and managed by the State of New York (as part of the Hudson River National Estuarine Research Reserve), there is a high potential for management activity to promote wetland resilience. As an example of how the analysis can inform management decisions, we used our geoprocessing tool to query the uncertainty analysis iterations for the likelihood of inundation of any type of wetland by year 2100 (Fig 10). The areas with the highest likelihood of inundation may guide management aimed at promoting accretion.

**Fig 11:**
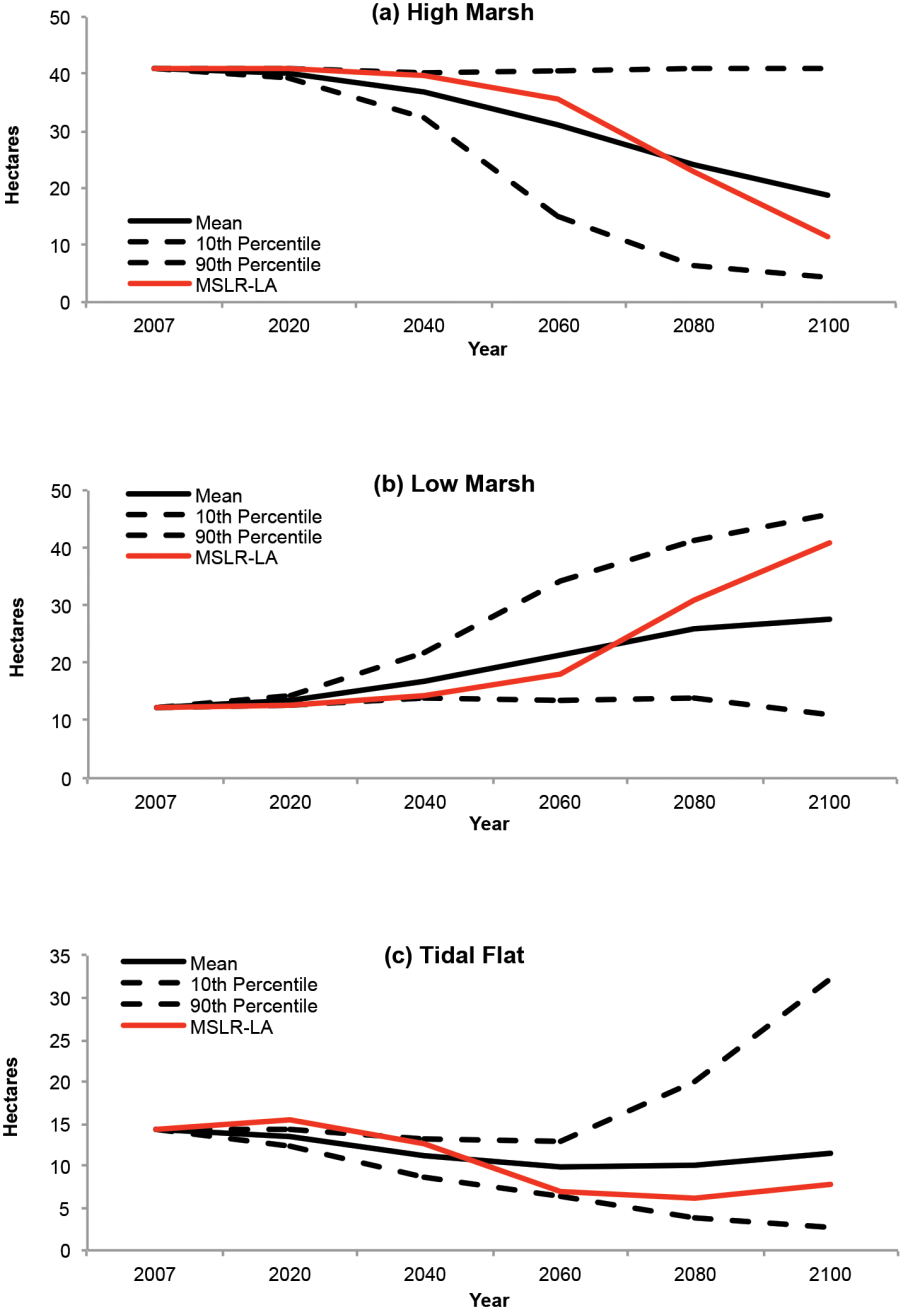
Comparison of the current trend scenario deterministic model (MSLR-LA) and uncertainty analysis results. Uncertainty analysis results include mean, 10^th^ percentile, and 90^th^ percentile for Iona Island Marsh’s (a) high marsh, (b) low marsh, and (c) tidal flat categories.

The uncertainty analysis results can be compared to the MSLR-LA deterministic model results by comparing the mean and range of values associated with the iterations. For Iona Island Marsh, the mean values of the uncertainty model iterations moderated the trends projected by the deterministic model over the century for each wetland type (Fig 11).

Some places where the uncertainty analysis iterations overlap most in wetland projections and resilience classification may reflect error in the original wetland class mapping. For instance, a high marsh area mis-classified as tidal flat would be projected by most model iterations to be resilient due to the one-way nature of the SLAMM decision tree (i.e., wetland classes can only convert towards classes lower in the tidal frame).

Given the uncertainty inherent in many of our model’s parameters, uncertainty analyses such as the one for Iona Island Marsh are important for, and translate well into, the design of effective site-specific land conservation and restoration efforts [75]. These analyses can also help inform where monitoring plots would be most effective for model verification and improvements.

## Conclusions

Our study represents the first comprehensive assessment of possible tidal wetland responses to sea level rise in the HRE. Based on its high local rates of SLR, microtidal environment, prevailing steep shoreline topography, a long history of shoreline development, as well as observed and projected tidal wetland losses around the globe, the HRE could be considered at high risk to wetland losses. However, our study’s findings indicate that there may be, in fact, considerable opportunity for inland wetland migration and localized wetland resilience through the 21^st^ century, owing in large part to the availability of low-gradient floodplains at the base of the steeper slopes in some parts of the estuary. If current rates of SLR do not increase dramatically, losses of existing wetland could amount to less than 15% of existing wetlands by the year 2100, which would be offset by considerably larger gains in new marsh; wetland composition may remain relatively stable under moderate or high accretion conditions.

However, there is also cause for great concern over the long-term fate of the HRE’s tidal wetlands. If high SLR rate projections are realized while accretion remains low, the projected net gains in wetlands could experience a steep decline late in the century. Under high rates of SLR there could be a dramatic shift in wetland habitat composition and wetland losses in the range of 21–60% of the current tidal wetland area. Even if the high sea levels are not experienced during this century, our simulations may offer a glimpse of tidal wetland fate beyond the 21^st^ century. Net losses in the longer term can be expected in any scenario where the rate of SLR outpaces accretion, since in most places in the estuary the low floodplains eventually meet with steeper slopes that will hinder marsh migration.

Our models necessarily simplify the very complex environment and processes of the HRE, and due to uncertainty in the projected rates of SLR and accretion it is possible that our results do not fully capture the possible magnitude of change in this system. By illustrating general trends in the primary geophysical processes and the availability of physically suitable future tidal habitat areas these results nevertheless represent a critical first step in regional conservation planning. The examination of projected trends and conserved lands by wetland system within the estuary is aimed at improving conservation practice by enabling an informed process of prioritization. Our analysis of projected changes in terms of wetland resilience to SLR represents an innovation that is also intended to inform future conservation and wetland management. Along with land conservation, ongoing work in the estuary is targeting shoreline and side channel restoration as well as invasive species control and native marsh restoration. Our study highlights the need for additional and higher quality site-specific data to inform such efforts, but also provides a useful, heretofore absent, planning tool that will help ensure the persistence of HRE tidal wetlands into the future. We hope that our methodology can also inform the development of improved tools for assessing freshwater tidal wetlands in other parts of the world, and ultimately advance the management of these valuable ecosystems.

## Supporting Information

S1 Appendix Detailed methodology. (DOCX)

S1 Fig Images of HRE tidal wetlands. (TIF)

S1 Table Hudson River Estuary tidal wetland systems. (PDF)

S2 Table SLAMM site parameters. (PDF)

S3 Table Hudson River Estuary SLAMM model results. (PDF)

S4 Table Wetland resilience metrics. (PDF)

S5 Table Wetland resilience metrics by wetland system for the current trend scenario. (PDF)
