# Supplementary material for: Simulating the Effects of Sea Level Rise on the Resilience and Migration of Tidal Wetlands along the Hudson River
Source: PLoS One. 2016 Apr 4;11(4):e0152437. doi: 10.1371/journal.pone.0152437 (PMC4820276; doi:10.1371/journal.pone.0152437)
Supplement: S1 Appendix — (DOCX) [file pone.0152437.s001.docx]

**S1 Appendix. Detailed methodology.**

This document provides detail about the assumptions of the Sea Level Affecting Marshes Model (SLAMM), its parameterization for our study area, and the input data sets which we assembled, created and/or edited for its implementation. We used ArcGIS (1) to process GIS files which are publicly available through the New York State GIS clearinghouse (<http://gis.ny.gov>), as individually referenced in this text. The S2 Table provides detailed model parameters by subsite.

**SLAMM**

We used version 6.2 of SLAMM (2) to simulate changes in tidal wetlands in the Hudson River Estuary (HRE). SLAMM has been used successfully to capture overall patterns of tidal wetland change in previous studies (3,4), however, when interpreting the results presented here, it is important to be mindful of its assumptions and limitations. Some overarching assumptions of SLAMM include:

- Tide range, accretion and the surrounding land use do not change over time
- Wetland classes transition sequentially regardless of the magnitude of SLR during a given time step
- Establishment of new wetlands is driven by geomorphological factors (i.e., inundation) while biological and ecological factors have negligible effects on it in the long-term
- Wetlands communities come into equilibrium with the level of inundation at each time step
- Mapped vegetation classes correspond directly with SLAMM classes that can be defined by specific elevation ranges in the tidal frame

More specific assumptions associated with our application of SLAMM to the HRE include:

- Non-tidal wetlands and undeveloped uplands convert to tidal wetlands in under the same rules
- The originally mapped tidal wetland types grouped into the three classes we used (high marsh, low marsh, tidal flat) accrete at the same rate and transition among classes similarly in response to inundation
- The simplified wetland classification sufficiently describes the projected changes for both the brackish and freshwater wetlands of the HRE, and thus any impact of increased salinity over time (particularly in tidal wetlands that are currently freshwater) is adequately represented by the results
- Accretion is negligible above the tidal influence (see the Accretion section below for further detail on assumptions associated with the accretion model)
- Soil saturation by the water table has negligible effects on tidal wetland migration and transition (i.e., we did not use the soil saturation function)
- Erosion rates are negligible (or adequately accounted for by the specified rate of accretion in tidal flats)
- Agricultural land uses, differences in substrate (e.g., dredge spoil versus naturally formed soils), the position of wetland in relation to the river’s mainstem (e.g., within rail road bays, around islands), and natural and built barriers to tidal hydrology have negligible impacts on tidal wetland response to SLR

Climate change has various impacts, many of which will combine with SLR to influence coastal ecosystems. At the time of our study the SLAMM model did not explicitly account for climate change-driven variations such as changes in temperature, precipitation or atmospheric CO_2_ concentrations. Stochastic natural events which can have large effects on coastal systems in the short-term (e.g., storm tides carrying heavy sediment loads), are also not simulated by SLAMM, leading to an underlying assumption that such events will have relatively no long-term impact. Most of these limitations are not specific to SLAMM, and different modeling approaches each have different strengths, weaknesses and assumptions depending on their focus (5).

We expect that the SLAMM software, which has already undergone several updates, will continue to be improved. Perhaps the most useful update for its application to freshwater tidal wetlands would be the implementation of a fully customizable decision tree and tidal wetland classes, including sub-tidal wetlands (shallows). The option to specify the Time Zero model simulation to project class changes in both downward and upward directions will improve its use as a corrective tool (in addition to its primary use as a test of model fit to data). Integration of a distance to channel function in the accretion model would also improve SLAMM’s performance, particularly in places like the HRE where large tributaries represent an important component of the system’s function. We also expect that the accuracy and availability of data specific to the HRE will improve, allowing future work to more fully utilize the existing functionalities of SLAMM or other predictive models.

**Input Data Sets**

Appendix Table 1. A summary of data sets used as inputs (or used to process the inputs) to SLAMM.

| **Dataset** | **Source** | **Resolution** | **Edits** | **Notes** |
| --- | --- | --- | --- | --- |
| Digital Elevation Model (DEM) | Photo Science Inc.(for NYS DEC)(2011/2012) | 1 m | Resampled to a 5-m resolution, calibrated to the Mean Tide Level (MTL = 0) of approximately 2007 using a model based on the Vertical Datum dataset | Derived from LiDAR, vertical accuracy = 0.15 m as verified by control points. |
| Vertical Datum | Stevens Institute of Technology (2013) | 10 m |  | Used to create a model of elevation for the study area relative to MTL |
| Slope | DEM dataset | 1 m |  | Calculated in degrees from DEM dataset using ArcGIS |
| Tidal Wetlands | Cornell Institute for Resource Information Sciences (for NYS DEC)(2007) | 5 m | Manually edited in ArcGIS to improve accuracy and extent, combined with Hudson River and Land Cover datasets, converted to a 5-m resolution raster | A polygon shapefile for use at 1:24,000 scale |
| Hudson River | Scenic Hudson | 5 m | Converted to a 5-m resolution raster | A mosaic of areas below MTL according to the DEM dataset and a river edge polygon file digitized based on orthophotos and the DEM; only used in areas outside of Tidal Wetlands dataset |
| Non-Tidal Land Cover | National Land Cover Database (2006) | 30 m | Reclassified all non-tidal wetland classes to upland developed/undeveloped, and defined any location with >25% impervious surface as developed upland | Only used in areas outside of the Tidal Wetlands and Hudson River datasets |

***Elevation***

High resolution LiDAR data was remotely collected along the HRE by Photo Science Inc. and processed into a Digital Elevation Model (DEM) for the New York State Department of Environmental Conservation in 2011/2012 (6). The original 1-meter resolution DEM raster dataset was resampled to a 5-m cell size. This data was calibrated to a Mean Tide Level (MTL) of zero using a model created by Scenic Hudson based on a vertical datum study by the Stevens Institute of Technology (7). In order to correlate temporally to the land cover data used in SLAMM, this dataset’s MTL value was adjusted using observed SLR rates at the New York Battery gauge to approximate year 2007 conditions (8).

***Land Cover***

The land cover dataset was created by combining tidal wetlands, land cover, and Hudson River extent data sets (9). Tidal wetlands were mapped based on 2007 conditions by the Cornell Institute for Resource Information Sciences for the Hudson River National Estuarine Research Reserve and New York State Department of Environmental Conservation (10), and represented the most accurate available information on tidal wetlands in the HRE (as compared with National Wetlands Inventory data or earlier maps of the HRE). Still, this dataset had some limitations. It was not comprehensively georeferenced (but rather referenced to a base map created in 1991 from USGS topographic maps, and intended for use at a 1:24,000 scale) nor comprehensively field verified, and thus displayed relatively significant, but inconsistent, spatial offsets in relation to orthophotos and LiDAR data. The extent of the original wetland mapping was also limited to areas below the 10 meter contour line on USGS topographical maps, or the first perceived barrier to tidal water movement, so that considerable areas of higher elevation tidal wetlands were apparently excluded in the original mapping.

In places of significant offsets in the mapped wetlands we manually adjusted the location of polygons in ArcGIS to gain better accuracy in relation to orthophotos. Some areas that appeared to be tidally inundated based on orthophotos and LiDAR data were added to the map. In order to further improve the coverage of the tidal wetland data set, we used the “Time Zero” simulation in SLAMM, which is a model simulation of existing conditions with no increase in sea level (2). Our Time Zero simulation resulted in a 28% increase over the mapped tidal wetland area and a total of ca. 2,800 ha; this area corresponds more closely with previously published figures for tidal wetland extent in the HRE (11,12). The wetland added in this simulation consisted primarily of high marsh in areas which, based on examination of high-resolution orthoimagery and elevation, does host tidal wetlands. The result of the Time Zero simulation is thus a hybrid of manual (mostly remote) mapping and an automated classification. And while this tool has undoubtedly introduced some error, overall it produced a more accurate and comprehensive general representation of the tidal wetlands, and thus we used it as our land cover input for subsequent simulations with SLR.

Appendix Table 2. Changes to mapped tidal wetland areas based on the SLAMM Time Zero simulation.

|  | Mapped Area (ha) | Time Zero Simulation (ha) | Net Change |
| --- | --- | --- | --- |
| Tidal Flat | 538 | 580 | + 7.8% |
| Low Marsh | 682 | 701 | + 2.8% |
| High Marsh | 980 | 1535 | + 56.6% |
| Total Wetland | 2,200 | 2,816 | + 28% |

Prior to the Time Zero simulation, the wetland data were combined with a raster of the extent of the Hudson River in order to more accurately represent the river’s extent beyond the mapped areas. This raster was in itself a composite of areas below MTL according to the DEM and a river edge file digitized based on orthophotos and elevation data. To include adjacent upland areas the data were further combined with the 2006 National Land Cover Database (NLCD)(13) coverage of the study area. All NLCD developed land categories except “Developed, Open Space” were classified as developed upland; all other categories were classified as undeveloped uplands. We also added any areas identified in the NLCD impervious surface data as being >25% impervious to the upland developed category. Non-tidal wetlands (e.g., woody wetlands or emergent herbaceous wetlands) were classified as undeveloped upland because they were very inaccurately represented in the NLCD data in areas adjacent to the Hudson River. In SLAMM version 6.2 and with our simplified wetland classes, non-tidal wetlands and uplands convert to tidal wetlands under the same rules, so this classification did not affect the resulting tidal wetland projections. The NLCD and impervious surface data were re-sampled from their original 30-m resolution (to 5-m resolution), and likely represent the least accurate component of the final composite land cover dataset.

Based on previous simulations, an elevation analysis in SLAMM (Appendix Figure 1), and the SLAMM wetland conversion decision tree, the originally mapped wetland categories were reclassified to SLAMM categories as described in Table 1 with the resulting elevational distributions shown in Fig 2. While this reclassification of wetland categories represents a significant simplification of both the mapped wetlands categories and the available SLAMM categories, the simplification was necessary for primarily two reasons: 1) several of the original wetland data classes are based on species or species assemblages, while SLAMM categories are defined largely by elevation ranges, and 2) SLAMM category conversions are based primarily on processes observed in coastal saltwater wetlands and some are dependent on having additional data inputs (e.g., freshwater influence, a salinity model) that were not available for our applicable to the tidal Hudson. The overlap in mapped wetland categories’ elevational ranges we found in the HRE is consistent with the less distinct plant community zonation typically found in tidal freshwater wetlands as compared with saltwater wetlands (14).


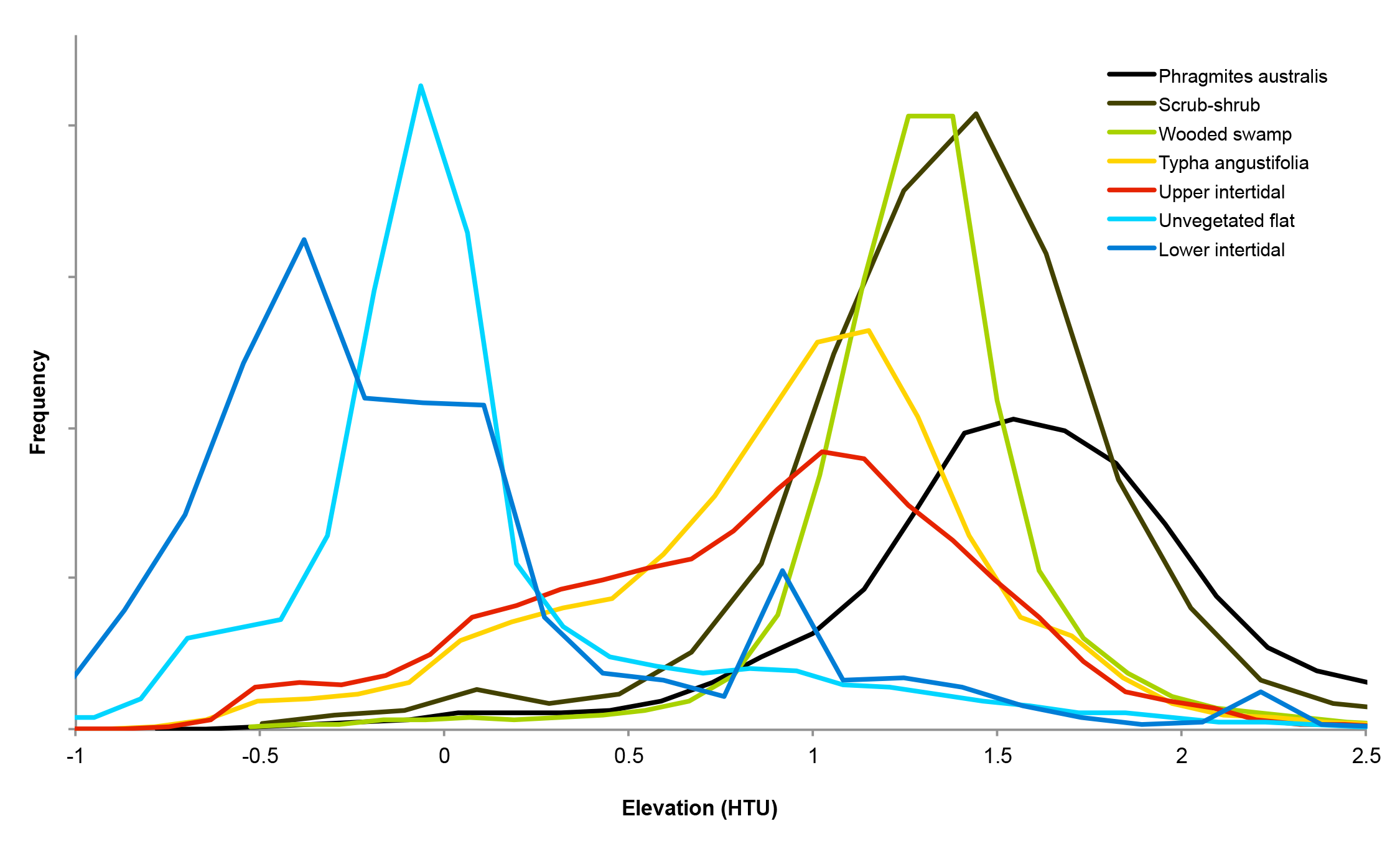
Appendix Figure 1. SLAMM elevation analysis of original wetland data classes from the north section of the HRE, showing the frequency of occurrence of each wetland class along an elevation gradient.

There were two instances when novel wetland classes were projected in the SLAMM outputs (i.e., classes that did not exist in the input data). When SLAMM projected the transition of upland to tidal wetland it was initially categorized as Transitional Salt Marsh; scrub/shrub wetlands, which correlate with this class, were lumped with high marsh in the input data (Table 1), and thus in our analysis of results all projected Transitional Salt Marsh areas were treated as part of the high marsh class. Under high erosion (as defined by a long fetch and adjacency to open water) and inundation conditions SLAMM projected the conversion of uplands to Estuarine Beach; these areas amounted to less than 0.5 ha for the entire estuary in any given simulation and had negligible impacts on the overall trends.

**Model Parameters**

***Sea Level Rise Projections***

We made two adjustments to the SLR projections for input into SLAMM. First, we used observed rates of SLR to calibrate the ClimAID projections (15), which are based on a 200-2004 starting time frame, to approximate our 2007 initial conditions date. Second, since SLAMM scales custom SLR rates to the A1B maximum scenario described by IPCC over a timeframe starting in 1990 (rather than the date of the simulation’s initial condition)(2) we adjusted the input projection values to achieve the desired resulting values by year 2100. Both of these adjustments are relatively minor, and likely imparted no significant effects on the study’s results.

Since the SLR projections were specific to our study site (i.e., they are local SLR projections, taking into account conditions such as subsidence and isostatic rebound), we set the local SLR historic trend parameter to 1.7 mm/year. This is the value that SLAMM version 6.2 uses as the eustatic SLR historic trend, which in turn is used to make adjustments between eustatic and user-specified local SLR rates (2). By setting the local trend to the same value as the eustatic trend we ensured no adjustments were made to our specified projections.

***Accretion***

Vertical accretion is critical to the adaptation of tidal wetlands to changes in sea level (16–21). Organic and mineral contributions to accretion likely vary by marsh elevation and salinity (14,20,21). While estuaries in the northeastern United States are sediment-poor compared with southeastern estuaries (17), the HRE has relatively high levels of suspended sediment including significant contributions from its major tributaries (22,23). However, sediment deposition rates can vary throughout the estuary (11) and are unlikely to be predictive of long term accretion rates without consideration of organic inputs and metabolic processes (18,24).

SLAMM version 6.2 incorporates functionality for describing the mechanistic relationship of accretion rates to elevation using simple models (2). We used a function roughly based on the application of the Marsh Equilibrium Model to the nearby New York City area (17,25) to create curves for low, medium, and high rates of accretion. The curves were defined by their maximum rates (5, 10 and 15 mm/year); a minimum accretion rate of zero; and cubic, quadratic, and linear model coefficients (-1.1, 0.6, 1, respectively). Using these curves, we derived the minimum and maximum rates of accretion for high marsh and low marsh based on their respective elevation ranges in our model (Appendix Table 3). In tidal flats we applied a constant rate of accretion of half the maximum low marsh accretion rate (26), which represents a gross estimate and a simplification of the more nuanced conditions that have been shown to exist in some freshwater tidal flats (20).

Appendix Table 3. Maximum and minimum accretion rates (in mm/year) used for the three levels of accretion in SLAMM model simulations.

| Accretion Level | Low | | Medium | | High | |
| --- | --- | --- | --- | --- | --- | --- |
|  | Max | Min | Max | Min | Max | Min |
| High Marsh | 4.3 | 1.9 | 8.6 | 3.9 | 12.9 | 5.9 |
| Low Marsh | 5 | 3.1 | 10 | 6.1 | 15 | 9.2 |
| Tidal Flat | 2.5 | 2.5 | 5 | 5 | 7.5 | 7.5 |

Our choice of three different accretion levels aimed to encompass the range of previously documented accretion rates in high land low marshes in the HRE (25,27–29). In our simulations the impact of differing rates of accretion was greatest when combined with high rates of SLR and during later time frames. The generic curves used in the accretion models describe a relative response of accretion rate to elevation change, but do not explicitly account for biotic factors that are known to have important impacts on accretion (17,30). For instance, different plant species and communities have been shown to have varied sediment trapping efficiencies and organic contributions to accretion (31,32). This phenomenon could have particularly strong implications in our tidal flat category, which combined vegetated and non-vegetated tidal flats. There is also likely to be variation in accretion at the scale of the entire estuary, with higher suspended sediment concentrations at the two primary Estuarine Turbidity Maxima (33,34). Additional differences across the HRE that likely affect rates of accretion are the position of the wetland in relation to railroad causeways (bay vs. open water), salinity, land uses in the adjacent watersheds and proximity to tributary mouths. This last factor may be accounted for in future updates of SLAMM through specifying a distance to stream channel in the model interface (2). We set all three of our generic curves to zero accretion at the elevation of 2 Half Tide Units (HTU). While tidally-related accretion does not take place above the tidal influence, the generic curves are not based on observed patterns in the HRE and this parameterization has implications for the wetland classes within the tidal frame (1.5 HTU and below) in our simulations. For high marsh in particular, the differing slopes in the three accretion curves likely had some influence on the projected rates of conversion to low marsh. Based on nearby New York City, Clough et al. modeled the accretion levels based on observed data to increase only slightly with decreasing elevation within the high marsh class (25), whereas our generic curves (and in particular the high accretion curve) resulted a more dramatic slope for this parameter. Given a lack of estuary-wide and cross elevational accretion data for the HRE at the time of our work, the generic curves allowed for a rough accounting of the mechanistic processes of accretion. We hope that future studies will enable us to more accurately model the accretion conditions across the HRE and its wetland types.

***Tide Ranges***

Larger tide ranges result in greater adaptability of tidal marshes to changes in sea level (19). The HRE is considered a microtidal system (with tide ranges < 2 meters), but the ranges vary considerably across the study site. We analyzed the tide range data from The Stevens Institute tidal datum model (7) to delineate 9 subsites based on breaks in the data when classified at 0.1 meter intervals, and used the median tide range in each subsite as the Great Diurnal Tide Range (GT) parameter (S2 Table). The wetland boundary elevation—or upper boundary between tidal and non-tidal environments—is inundated approximately once every 30 days, and is related to the Greater Diurnal Tide Range (GT). Based on studies in our region (25,35) the wetland boundary elevation for each subsite was approximated as 0.75 times the GT (or 1.5 HTU).

***Connectivity***

SLAMM version 6.2 provides an optional built-in connectivity algorithm, which prevents the model from projecting conversion to tidal wetland in low-lying areas that are not adjacent to existing tidal water or wetlands (2). This functionality was useful in our model simulations since our upland areas included non-tidal wetlands and gravel mines (i.e., artificially lowered elevation areas), which would otherwise be projected to convert to tidal wetland based on their elevation alone.

***Raster Data Analysis***

The resilience metrics (i.e., the analysis of changes in land cover and wetland types from Time Zero to year 2100 conditions) were quantified by using the Raster Calculator tool in the ArcGIS Spatial Analyst extension. Using this tool we calculated values that indicated, for each pixel in the resulting raster file, its condition in the input raster files. For instance, a given pixel with a Time Zero value of 8 (the ID number for low marsh) and a time 2100 value of 17 (the ID number for open water) was identified as a lost area of wetland by a raster calculation of (Time Zero value x 100) + 2100 value, and the resulting value of 817. Similar calculations that produce unique identifier values by raster pixel were used to related the projected wetland area to different land ownership categories (e.g., conserved vs. non-conserved), and to quantify the conflict of migrating wetlands with currently developed land uses.

***Uncertainty Analysis***

Performing an uncertainty analysis for the entire study area was complicated by the differing SLR rate projections for the north and south estuary sections, and computationally prohibitive in terms of processing time. Thus we performed uncertainty analyses on a selected subset of individual tidal wetland system areas. Using individual wetland systems as the study sites allowed us to run 500 simulations—a much higher number of iterations than we would be able to process on an estuary-wide analysis. We are able to continuously perform additional uncertainty analyses on wetland systems as needed to inform conservation decisions, and we also anticipate tailoring model parameters for wetlands where/when additional site-specific data are available (e.g., new site-specific accretion measures). Previous studies have shown that rate of SLR, accretion-related factors, and DEM error introduce the largest variability into SLAMM results (36), thus we varied the factors related to these parameters, as well as the tide range parameter. We assigned probability distributions to these parameters based on the MSLR-LA scenario (the current trend scenario) as described in Appendix Table 4.

Appendix Table 4. Model parameters and the probability distributions used in the wetland system-based uncertainty analyses.

| **Parameter** | **Distribution Type and Parameters** | **Notes** |
| --- | --- | --- |
| SLR by 2100 | Lognormal  Mean = 0.1  S.D. = 0.4 | The ClimAID SLR projections (provided in 10^th^, 25^th^-75^th^, and 90^th^ percentiles), approximate a lognormal distribution. We parameterized the mean and s.d. such that the most likely value approximates the input SLR projection (medium), while the standard deviation approximates 2.2 times this value falling in the 90th percentile (2.2 is the relative value of the 90^th^percentile projection to the input projection). |
| DEM Uncertainty | R.M.S.E = 0.19  Spatial A.C. = 0.2495 | The Root Mean Square Error (RMSE) of the DEM is 0.15 m, and the Mean Absolute Error (MAE) for MSL of the tidal datum data set is 0.041 m. The DEM data did not specify the level of autocorrelation. We used the sum of RMSE and MAE from the two data sets (0.19 m) and a high spatial auto correlation value of 0.2495 to account for maximum possible error. |
| Low Marsh and High Marsh Maximum Accretion | Triangular  Most Likely = 1  Minimum = 0.8  Maximum = 3 | The distribution reflects current knowledge of maximum accretion levels in the Hudson River Estuary. We varied only the maximum accretion to avoid iterations where a minimum exceeded a maximum and to maintain the general shape of the generic accretion curve. |
| Tidal Flat Maximum Accretion | Normal  Mean = 1  S.D. = 0.1 | This distribution allows for some variation in this parameter without significantly exceeding the range of possible accretion in high and low marsh categories. |
| Great Diurnal Tide Range | Normal  Mean = 1  S.D. = 0.03 | Based on the metadata of the tidal datum model, the MHHW Maximum Absolute Error (MAE)(0.024 m) and MLLW MAE (0.049 m) sum up to 0.073 m. This is 0.055 of the estuary’s mean tide range of 1.319 m. The standard deviation was set to approximate most values falling within this range of error, although the likelihood of the MAE for both MHHW and MLLW co-occurring at any given location is low. While the error varies depending on location in the estuary (and is generally higher in the northern portions), the metadata of the tidal datum model was not specific enough to customize a distribution to each subsite’s specific tidal range. |

**References**

1. ESRI Inc. ArcGIS Desktop. Redlands: Environmental Science Research Institute; 2013.

2. Clough J, Park RA, Propato M, Polaczyk A, Fuller R. SLAMM 6.2 Technical Documentation. Warren: Warren Pinnacle Consulting, Inc.; 2012.

3. Glick P, Clough J, Polaczyk A, Couvillion B, Nunley B. Potential Effects of Sea-Level Rise on Coastal Wetlands in Southeastern Louisiana. J Coast Res. 2013;Sp.Issue(63):211–33.

4. Geselbracht L, Freeman K, Kelly E, Gordon DR, Putz FE. Retrospective and prospective model simulations of sea level rise impacts on Gulf of Mexico coastal marshes and forests in Waccasassa Bay, Florida. Clim Change. 2011;107(1):35–57.

5. Mcleod E, Poulter B, Hinkel J, Reyes E, Salm R. Sea-level rise impact models and environmental conservation: A review of models and their applications. Ocean Coast Manag. 2010;53(9):507–17.

6. Photo Science Inc. Coastal New York LiDAR (Tidal Water Raster DEM). Albany: New York State Department of Environmental Conservation; 2012. Available from: http://gis.ny.gov/elevation/DEM-1-Meter-NOAA.htm

7. Georgas N. Hudson River Estuary Tidal Datums. Albany: New York State Department of Environmental Conservation; 2013. Available from: http://gis.ny.gov/gisdata/fileserver/?DSID=1136&file=TidalDatums.zip

8. Scenic Hudson Inc. HRE SLAMM 2007 DEM. Poughkeepsie: Scenic Hudson, Inc.; 2014. Available from: http://gis.ny.gov/gisdata/inventories/member.cfm?organizationID=696

9. Scenic Hudson Inc. HRE SLAMM Landcover. Poughkeepsie: Scenic Hudson, Inc.; 2014. Available from: http://gis.ny.gov/gisdata/inventories/member.cfm?organizationID=696

10. Cornell Institute for Resource Information Sciences. Hudson River Estuary Tidal Wetlands 2007. Albany: Hudson River National Estuarine Research Reserve and New York State Department of Environmental Conservation; 2011. Available from: http://gis.ny.gov/gisdata/fileserver/?DSID=1210&file=tidalwetlands2007.zip

11. Kiviat E, Findlay SEG, Nieder WC. Tidal Wetlands of the Hudson River Estuary. In: Levinton JS, Waldman JR, editors. The Hudson River Estuary. New York: Cambridge University Press; 2006. p. 279–95.

12. Strayer DL. The Hudson Primer: The Ecology of an Iconic River. London: University of California Press; 2012.

13. Fry J, Xian G, Jin S, Dewitz J, Homer C, Yang L, et al. Completion of the 2006 National Land Cover Database for the Conterminous United States. Photogramm Eng Remote Sens. 2011;77(9):858–64.

14. Odum WE. Comparative Ecology of Tidal Freshwater and Salt Marshes. Annu Rev Ecol Syst. 1988;19:147–76.

15. Horton RM, Bader D, Rosenzweig C, DeGaetano AT, Solecki W. Climate Change in New York State. Albany: New York State Energy Research and Development Authority; 2014.

16. Warren RS, Niering WA. Vegetation Change on a Northeast Tidal Marsh: Interaction of Sea-Level Rise and Marsh Accretion. Ecology. 1993;74(1):96–103.

17. Morris JT, Sundareshwar P V., Nietch CT, Kjerfve B, Cahoon DR. Responses of Coastal Wetlands to Rising Sea Level. Ecology. 2002;83(10):2869.

18. Neubauer SC, Anderson IC, Constantine JA, Kuehl SA. Sediment Deposition and Accretion in a Mid-Atlantic (U.S.A.) Tidal Freshwater Marsh. Estuarine, Coastal, Shelf Sci. 2002;54:713–27.

19. Kirwan ML, Guntenspergen GR, D’Alpaos A, Morris JT, Mudd SM, Temmerman S. Limits on the adaptability of coastal marshes to rising sea level. Geophys Res Lett. 2010;37(23):1–5.

20. Cadol D, Engelhardt K, Elmore A, Sanders G. Elevation-dependent surface elevation gain in a tidal freshwater marsh and implications for marsh persistence. Limnol Oceanogr. 2014;59(3):1065–80.

21. Schile LM, Callaway JC, Morris JT, Stralberg D, Thomas Parker V, Kelly M. Modeling tidal marsh distribution with sea-level rise: Evaluating the role of vegetation, sediment, and upland habitat in marsh resiliency. PLoS One. 2014;9(2).

22. Levinton JS, Waldman JR. The Hudson River Estuary: Executive Summary. In: Levinton JS, Waldman JR, editors. The Hudson River Estuary. New York: Cambridge University Press; 2006. p. 1–10.

23. Wall GR, Nystrom EA, Litten S. Suspended sediment transport in the freshwater reach of the Hudson river estuary in eastern New York. Estuaries and Coasts. 2008;31(3):542–53.

24. Neubauer SC, Craft CB. Global Change and Tidal Freshwater Wetlands: Scenarios and Impacts. In: Barendregt A, Whigham D, Baldwin A, editors. Tidal Freshwater Wetlands. Leiden: Backhuys Publishers; 2009. p. 253–66.

25. Clough J, Polaczyk A, Propato M. Application of Sea-Level Affecting Marshes Model (SLAMM) to Long Island , NY and New York City. Albany: New York State Energy Research and Development Authority; 2014.

26. Park RA, Lee JK, Canning DJ. Potential Effects of Sea-Level Rise on Puget Sound Wetlands. Geocarto Int. 1993;8(4):99–110.

27. Benoit G, Wang EX, Nieder WC, Levandowsky M, Breslin VT. Sources and history of heavy metal contamination and sediment deposition in Tivoli South Bay, Hudson River, New York. Estuaries. 1999;22(2A):167–78.

28. Pederson DC, Peteet DM, Kurdyla D, Guilderson T. Medieval Warming, Little Ice Age, and European impact on the environment during the last millennium in the lower Hudson Valley, New York, USA. Quat Res. 2005;63(3):238–49.

29. Sritrairat S, Peteet DM, Kenna TC, Sambrotto R, Kurdyla D, Guilderson T. A history of vegetation, sediment and nutrient dynamics at Tivoli North Bay, Hudson Estuary, New York. Estuar Coast Shelf Sci. 2012;102-103:24–35.

30. Kirwan ML, Murray AB. A coupled geomorphic and ecological model of tidal marsh evolution. Proc Natl Acad Sci U S A. 2007;104(15):6118–22.

31. Li H, Yang SL. Trapping Effect of Tidal Marsh Vegetation on Suspended Sediment, Yangtze Delta. J Coast Res. 2009;254:915–24.

32. Rooth JE, Stevenson JC, Cornwell JC. Increased sediment accretion rates following invasion by Phragmites australis: The role of litter. Estuaries. 2003;26(2):475–83.

33. Bokuniewicz H. Sedimentary processes in the Hudson River Estuary. In: Levinton JS, Waldman JR, editors. The Hudson River Estuary. New York: Cambridge University Press; 2006. p. 39–50.

34. Baldwin AH, Barendregt A, Whigham DF. Tidal Freshwater Wetlands: An Introduction to the Ecosystem. In: Barendregt A, Whigham D, Baldwin A, editors. Tidal freshwater wetlands. Leiden: Backhuys Publishers; 2009. p. 1–10.

35. Clough J, Polaczyk A, Propato M. Application of SLAMM to Coastal Connecticut: Final Report. Lowell: New England Interstate Water Pollution Control Commission; 2015.

36. Chu-Agor ML, Muñoz-Carpena R, Kiker G, Emanuelsson A, Linkov I. Exploring vulnerability of coastal habitats to sea level rise through global sensitivity and uncertainty analyses. Environ Model Softw. 2011;26(5):593–604.
