# Supplementary figures and images for: Simulating the Effects of Sea Level Rise on the Resilience and Migration of Tidal Wetlands along the Hudson River
Source: PLoS One. 2016 Apr 4;11(4):e0152437. doi: 10.1371/journal.pone.0152437 (PMC4820276; doi:10.1371/journal.pone.0152437)

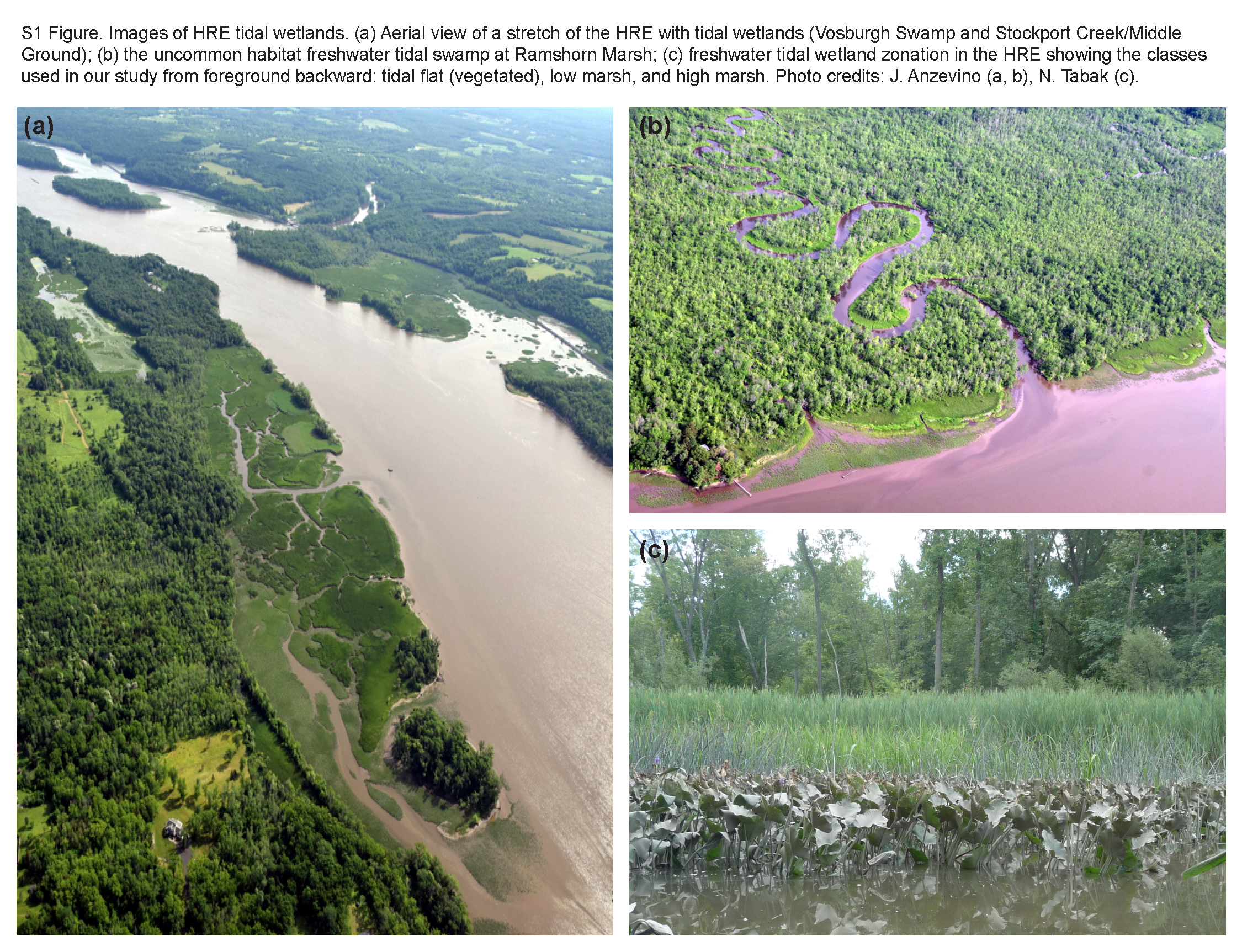

Supplement: S1 Fig — (TIF) [file pone.0152437.s002.tif]
