# Supplementary material for: Simulating the Effects of Sea Level Rise on the Resilience and Migration of Tidal Wetlands along the Hudson River
Source: PLoS One. 2016 Apr 4;11(4):e0152437. doi: 10.1371/journal.pone.0152437 (PMC4820276; doi:10.1371/journal.pone.0152437)
Supplement: S1 Table — (PDF) [file pone.0152437.s003.pdf]

S1 Table. Hudson River Estuary tidal wetland systems. Wetlands are ordered from North to South. Hectares are based on the Time Zero model simulation for the year 2007.

ID	Name (Alternate/Additional Names)	Hectares	Salinity Type
1	Lower Patroon & Breaker Islands	36	freshwater
2	Patroon Creek	14	freshwater
3	Hudson Mat	2	freshwater
4	Normans Kill Mouth	4	freshwater
5	Papscanee & Campbell Islands	166	freshwater
6	Binnen Kill (Shad & Schermerhorn Islands)	111	freshwater
7	Schodack/Houghtaling	280	freshwater
8	Hannacroix & Coeymans Creeks	27	freshwater
9	Mill Creek	71	freshwater
10	Coxsackie Creek & Bronck Island	28	freshwater
11	Coxsackie Shore & Islands (Rattlesnake Island)	31	freshwater
12	Nutten Hook/Gays Point/Stuyvesant Marsh	112	freshwater
13	Stockport Creek/Middle Ground	119	freshwater
14	Vosburgh Swamp (Four Mile Point)	82	freshwater
15	Athens Shore	8	freshwater
16	Middle Ground Flats	31	freshwater
17	Hudson North Bay	55	freshwater
18	Hudson South Bay (South Bay Creek & Marsh)	28	freshwater
19	Brandow Point	65	freshwater
20	Rogers Island	124	freshwater
21	Ramshorn Marsh/Inbocht Bay (Duck Cove, Catskill Creek)	380	freshwater
22	Roeliff Jansen Kill Mouth	13	freshwater
23	Esopus Creek Mouth	58	freshwater
24	Tivoli Bays (North & South Tivoli Bays)	177	freshwater
25	Rondout Creek Mouth	45	freshwater
26	Vanderburgh Cove	30	freshwater
27	Indian Kill Mouth	7	freshwater
28	Black Creek Mouth	3	freshwater
29	Maritje Kill/Crum Elbow	9	freshwater
30	Cedar Cliff	15	freshwater
31	Wappingers Creek Mouth	5	freshwater
32	Fishkill Creek/Dennings Point	9	freshwater
33	Moodna Creek Mouth	25	freshwater
34	Storm King	1	freshwater
35	Constitution Marsh	112	brackish
36	Con Hook	5	brackish
37	Manitou Marsh	25	brackish
38	Iona Island Marsh	68	brackish
39	Annsville Creek Mouth	15	brackish
40	Jones Point	0	brackish
41	Lents Cove	5	brackish
42	Georges Island	15	brackish
43	Furnace Brook/Oscawana Island	9	brackish
44	Stony Point	6	brackish
45	Minisceongo Creek Mouth	51	brackish
46	Croton (Croton River & Bay)	70	brackish
47	Pocantico River Mouth	1	brackish
48	Piermont Marsh	109	brackish
