# Supplementary material for: Simulating the Effects of Sea Level Rise on the Resilience and Migration of Tidal Wetlands along the Hudson River
Source: PLoS One. 2016 Apr 4;11(4):e0152437. doi: 10.1371/journal.pone.0152437 (PMC4820276; doi:10.1371/journal.pone.0152437)
Supplement: S2 Table — (PDF) [file pone.0152437.s004.pdf]

S2 Table. SLAMM site parameters.

	North Section					
Parameter	Global	SubSite 1	SubSite 2	SubSite 3	SubSite 4	SubSite 5
NWI Photo Date (YYYY)	2007	2007	2007	2007	2007	2007
DEM Date (YYYY)	2007	2007	2007	2007	2007	2007
Direction Offshore [n,s,e,w]	South	South	South	South	South	South
Historic Trend (mm/yr)	1.7	1.7	1.7	1.7	1.7	1.7
MTL-NAVD88 (m)	0	0	0	0	0	0
GT Great Diurnal Tide Range (m)	1.45	1.65	1.55	1.45	1.35	1.25
Salt Elev. (m above MTL)	1.088	1.238	1.163	1.088	1.013	0.938
Use Elev Pre-processor [True,False]	FALSE	FALSE	FALSE	FALSE	FALSE	FALSE
Reg Flood Use Model [True,False]	FALSE	FALSE	FALSE	FALSE	FALSE	FALSE
Reg Flood Max. Accr. (mm/year)*	5/10/15	5/10/15	5/10/15	5/10/15	5/10/15	5/10/15
Reg Flood Min. Accr. (mm/year)*	3.1/6.1/9.2	3.1/6.1/9.2	3.1/6.1/9.2	3.1/6.1/9.2	3.1/6.1/9.2	3.1/6.1/9.2
Reg Flood Elev a coeff. (cubic)	-1.1	-1.1	-1.1	-1.1	-1.1	-1.1
Reg Flood Elev b coeff. (square)	0.6	0.6	0.6	0.6	0.6	0.6
Reg Flood Elev c coeff. (linear)	1	1	1	1	1	1
Reg Flood D.Effect Max (meters)	0	0	0	0	0	0
Reg Flood D min. (unitless)	1	1	1	1	1	1
Reg Flood Salinity Turb. Max (ppt)	0	0	0	0	0	0
Reg Flood Turb. Max Zone (ppt)	0	0	0	0	0	0
Reg Flood S. Non T.Max (unitless)	1	1	1	1	1	1
Reg Flood Notes						
Irreg Flood Use Model [True,False]	FALSE	FALSE	FALSE	FALSE	FALSE	FALSE
Irreg Flood Max. Accr. (mm/year)*	4.3/8.6/12.9	4.3/8.6/12.9	4.3/8.6/12.9	4.3/8.6/12.9	4.3/8.6/12.9	4.3/8.6/12.9
Irreg Flood Min. Accr. (mm/year)*	1.9/3.9/5.9	1.9/3.9/5.9	1.9/3.9/5.9	1.9/3.9/5.9	1.9/3.9/5.9	1.9/3.9/5.9
Irreg Flood Elev a coeff. (cubic)	0	0	0	0	0	0
Irreg Flood Elev b coeff. (square)	0	0	0	0	0	0
Irreg Flood Elev c coeff. (linear)	1	1	1	1	1	1
Irreg Flood D.Effect Max (meters)	0	0	0	0	0	0
Irreg Flood D min. (unitless)	1	1	1	1	1	1
Irreg Flood Salinity Turb. Max (ppt)	0	0	0	0	0	0
Irreg Flood Turb. Max Zone (ppt)	0	0	0	0	0	0
Irreg Flood S. Non T.Max (unitless)	1	1	1	1	1	1
Irreg Flood Notes						
T.Flat Use Model [True,False]	FALSE	FALSE	FALSE	FALSE	FALSE	FALSE
T.Flat Max. Accr. (mm/year)*	2.5/5/7.5	2.5/5/7.5	2.5/5/7.5	2.5/5/7.5	2.5/5/7.5	2.5/5/7.5
T.Flat Min. Accr. (mm/year)*	2.5/5/7.5	2.5/5/7.5	2.5/5/7.5	2.5/5/7.5	2.5/5/7.5	2.5/5/7.5
T.Flat Elev a coeff. (cubic)	0	0	0	0	0	0
T.Flat Elev b coeff. (square)	0	0	0	0	0	0
T.Flat Elev c coeff. (linear)	0	0	0	0	0	0
T.Flat D.Effect Max (meters)	0	0	0	0	0	0
T.Flat D min. (unitless)	1	1	1	1	1	1
T.Flat Salinity Turb. Max (ppt)	0	0	0	0	0	0
T.Flat Turb. Max Zone (ppt)	0	0	0	0	0	0
T.Flat S. Non T.Max (unitless)	1	1	1	1	1	1
T.Flat Notes						

* The three values represent low/medium/high accretion models, respectively.

	South Section					
Parameter	Global	SubSite 1	SubSite 2	SubSite 3	SubSite 4	SubSite 5
NWI Photo Date (YYYY)	2007	2007	2007	2007	2007	2007
DEM Date (YYYY)	2007	2007	2007	2007	2007	2007
Direction Offshore [n,s,e,w]	South	South	South	South	South	South
Historic Trend (mm/yr)	1.7	1.7	1.7	1.7	1.7	1.7
MTL-NAVD88 (m)	0	0	0	0	0	0
GT Great Diurnal Tide Range (m)	1.174	1.25	1.15	1.07	1.15	1.25
Salt Elev. (m above MTL)	0.881	0.938	0.863	0.803	0.863	0.938
Use Elev Pre-processor [True,False]	FALSE	FALSE	FALSE	FALSE	FALSE	FALSE
Reg Flood Use Model [True,False]	FALSE	FALSE	FALSE	FALSE	FALSE	FALSE
Reg Flood Max. Accr. (mm/year)*	5/10/15	5/10/15	5/10/15	5/10/15	5/10/15	5/10/15
Reg Flood Min. Accr. (mm/year)*	3.1/6.1/9.2	3.1/6.1/9.2	3.1/6.1/9.2	3.1/6.1/9.2	3.1/6.1/9.2	3.1/6.1/9.2
Reg Flood Elev a coeff. (cubic)	-1.1	-1.1	-1.1	-1.1	-1.1	-1.1
Reg Flood Elev b coeff. (square)	0.6	0.6	0.6	0.6	0.6	0.6
Reg Flood Elev c coeff. (linear)	1	1	1	1	1	1
Reg Flood D.Effect Max (meters)	0	0	0	0	0	0
Reg Flood D min. (unitless)	1	1	1	1	1	1
Reg Flood Salinity Turb. Max (ppt)	0	0	0	0	0	0
Reg Flood Turb. Max Zone (ppt)	0	0	0	0	0	0
Reg Flood S. Non T.Max (unitless)	1	1	1	1	1	1
Reg Flood Notes						
Irreg Flood Use Model [True,False]	FALSE	FALSE	FALSE	FALSE	FALSE	FALSE
Irreg Flood Max. Accr. (mm/year)*	4.3/8.6/12.9	4.3/8.6/12.9	4.3/8.6/12.9	4.3/8.6/12.9	4.3/8.6/12.9	4.3/8.6/12.9
Irreg Flood Min. Accr. (mm/year)*	1.9/3.9/5.9	1.9/3.9/5.9	1.9/3.9/5.9	1.9/3.9/5.9	1.9/3.9/5.9	1.9/3.9/5.9
Irreg Flood Elev a coeff. (cubic)	0	0	0	0	0	0
Irreg Flood Elev b coeff. (square)	0	0	0	0	0	0
Irreg Flood Elev c coeff. (linear)	1	1	1	1	1	1
Irreg Flood D.Effect Max (meters)	0	0	0	0	0	0
Irreg Flood D min. (unitless)	1	1	1	1	1	1
Irreg Flood Salinity Turb. Max (ppt)	0	0	0	0	0	0
Irreg Flood Turb. Max Zone (ppt)	0	0	0	0	0	0
Irreg Flood S. Non T.Max (unitless)	1	1	1	1	1	1
Irreg Flood Notes						
T.Flat Use Model [True,False]	FALSE	FALSE	FALSE	FALSE	FALSE	FALSE
T.Flat Max. Accr. (mm/year)*	2.5/5/7.5	2.5/5/7.5	2.5/5/7.5	2.5/5/7.5	2.5/5/7.5	2.5/5/7.5
T.Flat Min. Accr. (mm/year)*	2.5/5/7.5	2.5/5/7.5	2.5/5/7.5	2.5/5/7.5	2.5/5/7.5	2.5/5/7.5
T.Flat Elev a coeff. (cubic)	0	0	0	0	0	0
T.Flat Elev b coeff. (square)	0	0	0	0	0	0
T.Flat Elev c coeff. (linear)	0	0	0	0	0	0
T.Flat D.Effect Max (meters)	0	0	0	0	0	0
T.Flat D min. (unitless)	1	1	1	1	1	1
T.Flat Salinity Turb. Max (ppt)	0	0	0	0	0	0
T.Flat Turb. Max Zone (ppt)	0	0	0	0	0	0
T.Flat S. Non T.Max (unitless)	1	1	1	1	1	1
T.Flat Notes						

* The three values represent low/medium/high accretion models, respectively.
