# Supplementary material for: Simulating the Effects of Sea Level Rise on the Resilience and Migration of Tidal Wetlands along the Hudson River
Source: PLoS One. 2016 Apr 4;11(4):e0152437. doi: 10.1371/journal.pone.0152437 (PMC4820276; doi:10.1371/journal.pone.0152437)
Supplement: S3 Table — (PDF) [file pone.0152437.s005.pdf]

S3 Table. Hudson River Estuary SLAMM model results (in hectares). Transitional Salt Marsh and Irregularly Flooded Marsh correspond with our High Marsh class, and Regularly Flooded Marsh with our Low Marsh.

	**SLR**	**Low**														
	**Accretion**	**Low**					**Medium**					**High**				
**Class**	**2007 (time 0)**	**2020**	**2040**	**2060**	**2080**	**2100**	**2020**	**2040**	**2060**	**2080**	**2100**	**2020**	**2040**	**2060**	**2080**	**2100**
undeveloped upland	15050.0	15021.1	14963.7	14879.7	14775.7	14663.6	15021.4	14964.6	14880.9	14777.9	14668.3	15025.4	14982.2	14918.5	14828.9	14738.6
Transitional Salt Marsh		28.7	85.9	169.2	270.2	374.2	28.4	85.0	168.2	270.2	375.2	24.4	67.6	131.1	219.6	307.3
Regularly Flooded Marsh	701.1	808.9	790.9	792.8	796.0	804.6	794.3	783.9	784.8	785.9	790.3	781.4	774.3	774.6	775.8	778.3
Estuarine Beach		0.2	0.2	0.2	0.2	0.2	0.2	0.2	0.2	0.2	0.2	0.2	0.2	0.2	0.2	0.2
Tidal Flat	579.8	597.5	604.5	596.9	590.2	577.8	594.6	598.0	597.0	597.0	597.1	592.3	594.7	594.5	594.5	594.6
Estuarine Water	27750.1	27752.0	27754.2	27759.9	27766.4	27778.1	27751.6	27752.1	27752.4	27752.4	27752.4	27751.2	27751.2	27751.3	27751.3	27751.3
Irregularly Flooded Marsh	1534.8	1407.4	1416.4	1417.1	1417.2	1417.3	1425.3	1432.1	1432.3	1432.3	1432.3	1441.0	1445.6	1445.6	1445.6	1445.6
**Totals**																
Total tidal wetland	2815.7	2842.7	2897.9	2976.2	3073.8	3174.1	2842.8	2899.1	2982.5	3085.5	3195.1	2839.2	2882.4	2946.1	3035.7	3126.0
Total high marsh	1534.8	1436.1	1502.3	1586.3	1687.5	1791.4	1453.7	1517.1	1600.5	1702.5	1807.5	1465.3	1513.2	1576.8	1665.2	1752.9
New open water		1.9	4.1	9.8	16.3	28.0	1.5	2.0	2.3	2.3	2.3	1.1	1.1	1.2	1.2	1.2

	**SLR**	**Medium**														
	**Accretion**	**Low**					**Medium**					**High**				
**Class**	**2007 (time 0)**	**2020**	**2040**	**2060**	**2080**	**2100**	**2020**	**2040**	**2060**	**2080**	**2100**	**2020**	**2040**	**2060**	**2080**	**2100**
undeveloped upland	15050.0	14963.8	14765.8	14454.4	14155.4	13848.2	14964.1	14767.0	14453.6	14154.1	13846.0	14964.5	14768.3	14451.2	14148.5	13843.6
Transitional Salt Marsh		86.0	279.4	557.3	648.4	602.7	85.7	278.8	563.9	758.2	788.2	85.3	280.0	587.7	842.9	1055.6
Regularly Flooded Marsh	701.1	822.2	839.6	1056.9	1649.6	2242.9	812.9	807.4	895.2	1085.8	1377.4	790.5	781.9	791.3	838.5	929.7
Estuarine Beach		0.2	0.2	0.2	0.2	0.2	0.2	0.2	0.2	0.2	0.2	0.2	0.2	0.2	0.2	0.2
Tidal Flat	579.8	591.6	591.4	543.3	483.0	483.6	593.2	587.0	542.0	491.1	445.6	593.7	596.5	591.7	573.8	540.9
Estuarine Water	27750.1	27761.1	27805.3	27902.3	28034.5	28137.6	27756.6	27786.0	27851.1	27931.7	28029.1	27751.7	27752.4	27756.9	27775.1	27808.8
Irregularly Flooded Marsh	1534.8	1391.0	1334.3	1101.4	644.7	300.6	1403.1	1389.5	1309.9	1194.7	1129.3	1430.0	1436.6	1436.7	1436.8	1436.9
**Totals**																
Total tidal wetland	2815.7	2890.9	3044.7	3259.1	3425.9	3630.0	2895.1	3062.8	3311.1	3530.1	3740.8	2899.6	3095.1	3407.7	3692.2	3963.4
Total high marsh	1534.8	1476.9	1613.6	1658.6	1293.1	903.3	1488.8	1668.3	1873.7	1952.9	1917.5	1515.3	1716.5	2024.4	2279.8	2492.6
New open water		11.0	55.2	152.2	284.4	387.5	6.5	35.9	101.0	181.6	279.0	1.6	2.3	6.8	25.0	58.7

	**SLR**	**High**														
	**Accretion**	**Low**					**Medium**					**High**				
**Class**	**2007 (time 0)**	**2020**	**2040**	**2060**	**2080**	**2100**	**2020**	**2040**	**2060**	**2080**	**2100**	**2020**	**2040**	**2060**	**2080**	**2100**
undeveloped upland	15050.0	14850.1	14348.3	13787.1	13273.7	12832.6	14850.6	14347.9	13786.7	13274.5	12832.3	14851.2	14347.3	13786.2	13274.6	12831.9
Transitional Salt Marsh		199.7	610.8	572.1	491.1	421.4	199.2	640.3	615.3	498.2	415.9	198.6	662.2	676.2	526.0	428.7
Regularly Flooded Marsh	701.1	887.8	1472.9	2082.0	1566.8	1022.7	836.1	1165.2	2362.7	2479.8	1818.3	810.3	871.3	2056.0	3128.4	3271.6
Estuarine Beach		0.2	0.2	0.2	0.1	0.1	0.2	0.2	0.2	0.1	0.1	0.2	0.2	0.2	0.1	0.1
Tidal Flat	579.8	586.3	551.4	758.6	1611.8	1790.3	581.6	518.1	455.1	895.1	1831.9	585.0	514.4	369.7	273.7	643.4
Estuarine Water	27750.1	27790.7	28002.7	28274.9	28601.3	29501.8	27775.7	27931.2	28156.4	28377.5	28661.6	27764.1	27855.7	28062.8	28263.6	28368.0
Irregularly Flooded Marsh	1534.8	1301.1	629.5	140.8	71.0	46.9	1372.4	1013.0	239.4	90.7	55.8	1406.4	1364.6	664.8	149.5	72.2
**Totals**																
Total tidal wetland	2815.7	2975.0	3264.8	3553.8	3740.8	3281.4	2989.5	3336.7	3672.7	3963.8	4121.9	3000.5	3412.8	3766.8	4077.7	4415.8
Total high marsh	1534.8	1500.8	1240.3	712.9	562.1	468.3	1571.6	1653.3	854.7	588.9	471.7	1605.0	2026.9	1340.9	675.5	500.9
New open water		40.5	252.6	524.8	851.2	1751.7	25.6	181.1	406.3	627.4	911.5	14.0	105.6	312.7	513.5	617.9
