# Supplementary material for: Simulating the Effects of Sea Level Rise on the Resilience and Migration of Tidal Wetlands along the Hudson River
Source: PLoS One. 2016 Apr 4;11(4):e0152437. doi: 10.1371/journal.pone.0152437 (PMC4820276; doi:10.1371/journal.pone.0152437)
Supplement: S4 Table — (PDF) [file pone.0152437.s006.pdf]

S4 Table. Wetland resilience metrics (by year 2100, in hectares). The most resilient wetlands are those that remain in the same tidal wetland class; somewhat resilient wetlands change in class but remain a type of tidal wetland.

	Scenario					
**Resilience Metric**	**MSLR-LA**	**MSLR-MA**	**MSLR-HA**	**HSLR-LA**	**HSLR-MA**	**HSLR-HA**
**New wetland, previously developed upland**	329	336	340	804	836	848
**New wetland, previously undeveloped upland**	1202	1206	1206	2163	2206	2214
**Somewhat resilient wetland**	1390	236	109	1019	1740	1676
**Most resilient wetland**	1038	2342	2648	100	177	526
**Lost wetland**	387	238	59	1697	899	614
