# Supplementary material for: Simulating the Effects of Sea Level Rise on the Resilience and Migration of Tidal Wetlands along the Hudson River
Source: PLoS One. 2016 Apr 4;11(4):e0152437. doi: 10.1371/journal.pone.0152437 (PMC4820276; doi:10.1371/journal.pone.0152437)
Supplement: S5 Table — (PDF) [file pone.0152437.s007.pdf]

S5 Table. Wetland resilience metrics by wetland system: medium SLR, low accretion, year 2100 (in hectares). The most resilient wetlands are those that remain in the same tidal wetland class; somewhat resilient wetlands change in class but remain a type of tidal wetland.

**Wetland ID**	1	2	3	4	5	6	7	8	9	10	11	12
**Wetland Name**	**Lower Patroon & Breaker Islands**	**Patroon Creek**	**Hudson Mat**	**Normans Kill Mouth**	**Papscanee & Campbell Islands**	**Binnen Kill**	**Schodack/ Houghtaling**	**Hannacroix & Coeymans Creeks**	**Mill Creek**	**Coxsackie Creek & Bronck Island**	**Coxsackie Shore & Islands**	**Nutten Hook/Gays Point/ Stuyvesant Marsh**
most resilient wetland, protected	0.1	0.0	0.0	0.0	7.4	39.3	72.0	0.9	7.7	6.9	0.4	46.0
most resilient wetland, unprotected	30.8	5.4	0.2	0.7	47.3	14.0	55.7	12.0	19.1	11.4	9.1	10.8
somewhat resilient wetland, protected	0.2	0.0	0.0	0.0	26.2	34.3	123.4	1.3	24.2	3.3	1.1	50.1
somewhat resilient wetland, unprotected	4.8	8.4	1.6	3.1	82.6	22.6	24.5	11.5	20.3	5.7	15.9	3.0
new wetland, protected, undeveloped	0.8	0.0	0.0	0.0	89.6	137.9	77.9	5.0	3.2	9.5	1.9	47.0
new wetland, unprotected, undeveloped	18.0	6.3	0.8	5.2	175.1	33.5	29.6	20.6	14.0	12.5	17.7	4.2
new wetland, protected, developed	0.3	0.0	0.0	0.0	2.4	0.4	1.1	0.0	0.4	0.0	0.4	0.8
new wetland, unprotected, developed	1.2	0.0	1.2	2.1	11.9	0.8	3.9	2.9	2.0	0.0	1.6	3.3
wetlands lost, protected	0.0	0.0	0.0	0.0	0.0	0.5	0.5	0.0	0.1	0.0	0.1	2.2
wetlands lost, unprotected	0.0	0.0	0.0	0.0	2.9	0.6	3.5	1.6	0.0	0.4	4.1	0.1
total wetlands (development excluded)	54.7	20.1	2.6	9.0	428.1	281.5	383.1	51.4	88.3	49.3	46.1	161.1

**Wetland ID**	13	14	15	16	17	18	19	20	21	22	23	24
**Wetland Name**	**Stockport Creek/ Middle Ground**	**Vosburgh Swamp**	**Athens Shore**	**Middle Ground Flats**	**Hudson North Bay**	**Hudson South Bay**	**Brandow Point**	**Rogers Island**	**Ramshorn Marsh/ Inbocht Bay**	**Roeliff Jansen Kill Mouth**	**Esopus Creek Mouth**	**Tivoli Bays**
most resilient wetland, protected	50.5	14.8	0.5	4.5	20.7	0.0	0.3	17.7	28.7	0.3	0.0	94.0
most resilient wetland, unprotected	11.5	35.6	0.6	0.3	10.6	9.9	28.6	5.1	60.3	4.6	14.0	2.1
somewhat resilient wetland, protected	46.2	5.1	0.8	18.3	12.5	0.1	0.5	48.0	103.4	0.3	0.1	39.8
somewhat resilient wetland, unprotected	7.4	15.4	2.9	0.2	4.4	18.0	24.2	6.4	135.7	6.0	24.3	1.8
new wetland, protected, undeveloped	17.1	7.2	1.1	29.2	2.0	0.1	0.8	12.8	13.0	0.6	0.1	6.6
new wetland, unprotected, undeveloped	10.5	7.3	3.6	0.0	1.0	21.8	3.5	1.0	33.0	3.1	6.1	0.8
new wetland, protected, developed	0.9	0.1	1.5	0.0	0.0	0.3	0.0	0.0	0.2	0.0	0.0	5.9
new wetland, unprotected, developed	2.0	1.4	3.3	0.0	1.5	4.0	1.8	0.0	1.2	1.4	6.4	0.8
wetlands lost, protected	1.9	0.0	1.0	5.9	5.2	0.0	0.0	20.3	6.2	0.4	0.0	34.3
wetlands lost, unprotected	1.9	11.6	1.8	2.3	1.3	0.3	11.9	26.3	46.1	1.9	19.6	5.2
total wetlands (development excluded)	143.3	85.3	9.5	52.5	51.1	50.0	57.9	91.0	374.1	14.7	44.6	145.0

Wetland ID	25	26	27	28	29	30	31	32	33	34	35	36
**Wetland Name**	**Rondout Creek Mouth**	**Vanderburgh Cove**	**Indian Kill Mouth**	**Black Creek Mouth**	**Maritje Kill/Crum Elbow**	**Cedar Cliff**	**Wappingers Creek Mouth**	**Fishkill Creek/ Dennings Point**	**Moodna Creek Mouth**	**Storm King**	**Constitution Marsh**	**Con Hook**
most resilient wetland, protected	1.6	1.8	0.9	0.3	0.7	0.0	0.0	0.2	7.0	0.0	45.8	2.9
most resilient wetland, unprotected	0.5	2.5	0.3	0.4	0.4	5.9	0.2	1.5	0.7	0.0	5.2	0.0
somewhat resilient wetland, protected	12.3	3.3	4.2	1.6	2.2	0.0	1.1	1.5	9.8	1.1	27.3	2.0
somewhat resilient wetland, unprotected	15.7	4.0	0.4	0.5	0.4	6.4	2.3	2.9	2.9	0.0	3.2	0.0
new wetland, protected, undeveloped	3.1	1.2	2.6	1.3	0.4	0.0	0.8	3.8	1.8	1.3	5.7	0.7
new wetland, unprotected, undeveloped	31.0	2.8	0.2	0.2	0.1	3.0	1.4	0.3	4.8	0.0	1.2	0.1
new wetland, protected, developed	2.2	0.0	0.3	0.0	0.0	0.0	0.2	0.1	0.1	0.0	0.5	0.0
new wetland, unprotected, developed	24.2	0.9	0.0	0.0	2.1	2.5	4.7	0.2	1.2	0.0	4.6	0.0
wetlands lost, protected	11.9	5.6	0.5	0.1	0.1	0.0	0.1	0.1	3.8	0.0	28.7	0.4
wetlands lost, unprotected	2.6	12.9	0.1	0.2	5.3	3.2	1.2	2.3	0.9	0.0	1.9	0.0
total wetlands (development excluded)	64.2	15.6	8.6	4.2	4.2	15.3	5.7	10.2	27.0	2.4	88.3	5.8

Wetland ID	37	38	39	40	41	42	43	44	45	46	47	48
**Wetland Name**	**Manitou Marsh**	**Iona Island Marsh**	**Annsville Creek Mouth**	**Jones Point**	**Lents Cove**	**Georges Island**	**Furnace Brook/ Oscawana Island**	**Stony Point**	**Minisceongo Creek Mouth**	**Croton**	**Pocantico River Mouth**	**Piermont Marsh**
most resilient wetland, protected	3.4	16.5	1.4	0.0	0.0	9.2	2.4	2.6	3.4	12.4	0.0	18.6
most resilient wetland, unprotected	15.0	0.0	5.3	0.0	1.1	0.0	0.0	0.2	5.2	8.3	0.0	0.2
somewhat resilient wetland, protected	4.5	35.6	1.8	0.1	0.2	6.0	1.9	2.2	5.7	15.7	1.0	83.7
somewhat resilient wetland, unprotected	1.4	0.0	4.1	0.1	1.7	0.0	0.0	1.3	25.2	16.9	0.3	2.2
new wetland, protected, undeveloped	1.6	4.8	2.8	0.6	0.2	2.2	0.7	0.6	2.5	9.1	1.7	2.9
new wetland, unprotected, undeveloped	1.3	0.0	5.5	0.7	0.9	0.0	0.1	0.1	19.7	4.2	0.5	2.1
new wetland, protected, developed	0.0	2.5	1.4	0.0	0.1	0.0	0.1	0.0	2.9	3.4	1.0	2.7
new wetland, unprotected, developed	0.2	0.0	10.0	0.2	0.4	0.0	0.0	4.9	27.0	6.4	4.2	12.4
wetlands lost, protected	0.5	15.6	0.1	0.0	0.0	0.0	4.5	0.1	3.4	0.8	0.1	4.1
wetlands lost, unprotected	0.6	0.0	2.8	0.0	1.6	0.0	0.0	0.1	8.3	16.3	0.0	0.3
total wetlands (development excluded)	27.0	56.9	21.0	1.6	4.1	17.4	5.1	7.0	61.6	66.6	3.4	109.7
